# Statistical inference of the time-varying structure of gene-regulation networks

**DOI:** 10.1186/1752-0509-4-130

**Published:** 2010-09-22

**Authors:** Sophie Lèbre, Jennifer Becq, Frédéric Devaux, Michael PH Stumpf, Gaëlle Lelandais

**Affiliations:** 1 Center for Bioinformatics, Imperial College London, London, UK; 2 Laboratoire des Sciences de l'Image de l'Informatique et de la télédétection (LSIIT), UMR UdS-CNRS 7005, Université de Strasbourg, Strasbourg, France; 3 Dynamique des Structures et Interactions des Macromolécules Biologiques (DSIMB), INSERM U 665, Paris, F-75015, France; 4 Université Paris Diderot - Paris 7, UMR-S665, Paris, F-75015, France; 5 INTS, Paris, F-75015, France; 6 Laboratoire de Génomique des Microorganismes, CNRS FRE 3214, Université Pierre et Marie Curie, Institut des Cordeliers, Paris, France; 7 Institute of Mathematical Sciences, Imperial College London, London, UK

## Abstract

**Background:**

Biological networks are highly dynamic in response to environmental and physiological cues. This variability is in contrast to conventional analyses of biological networks, which have overwhelmingly employed static graph models which stay constant over time to describe biological systems and their underlying molecular interactions.

**Methods:**

To overcome these limitations, we propose here a new statistical modelling framework, the ARTIVA formalism (Auto Regressive TIme VArying models), and an associated inferential procedure that allows us to learn temporally varying gene-regulation networks from biological time-course expression data. ARTIVA simultaneously infers the topology of a regulatory network and how it changes over time. It allows us to recover the chronology of regulatory associations for individual genes involved in a specific biological process (development, stress response, etc.).

**Results:**

We demonstrate that the ARTIVA approach generates detailed insights into the function and dynamics of complex biological systems and exploits efficiently time-course data in systems biology. In particular, two biological scenarios are analyzed: the developmental stages of *Drosophila melanogaster *and the response of *Saccharomyces cerevisiae *to benomyl poisoning.

**Conclusions:**

ARTIVA does recover essential temporal dependencies in biological systems from transcriptional data, and provide a natural starting point to learn and investigate their dynamics in greater detail.

## Background

Molecular interactions and regulatory networks underlie the development and functioning of biological systems [1-3]. These networks reliably and robustly coordinate the molecular and biochemical processes inside a cell, while remaining flexible in order to respond to physiological and environmental changes. The changing nature of regulatory and signalling interactions is beyond doubt, and a dynamical point of view is already deeply enshrined into cell and molecular biology. Illustrations of such time-varying biological systems can be provided for instance by the development of the fruitfly *Drosophila melanogaster *- which is segmented into different life stages: embryogenesis, larva, pupa and adult, or the adaptation of cellular organisms (the yeast *Saccharomyces cerevisiae *for instance) to growth defects and cellular damages induced by environmental stresses. Because they are extensively studied, considerable large-scale functional screening data exist for these examples. But while a growing number of studies report detailed and time-resolved analyses of regulatory and signalling processes [4,5], mapping these temporally changing networks systematically remains a major and increasingly pressing challenge.

From available data, *in-silico *methods can generate hypotheses about biochemical and molecular mechanisms [6,7] and guide further experimental and theoretical investigations into regulatory interactions underlying biological systems. Biological networks are usually described mathematically in a way where each gene is represented by a node and the interactions (or regulatory associations) between genes as edges. A range of approaches has been proposed, which learn or infer correlative and causal relationships among the genes from high-throughput, in particular gene expression, data. However most of these approaches assume that the topology of the network, *i.e*. the sets of nodes and edges, stays constant over time. Inferring the temporal changes in biological networks is an important statistical challenge [8], but it does open up new perspectives for biological data analyses and will aid the generation of hypotheses about the dynamics of biological systems.

Serious attempts to reconstruct dynamic networks whose topology changes with time started in 2005 [9,10]. Yoshida *et al*. [9] employed a dynamic linear model with Markov switching for estimating time-dependent gene network structures from time-series gene expression data. Although promising this approach assumes that there is a fixed (user-specified) number of distinct networks or phases, and the switching between phases is modelled *via *a stochastic transition matrix that requires an estimation of many parameters. Talih and Hengarten [10] developed a Markov Chain Monte Carlo (MCMC) methodology to recover time-varying Gaussian graphical models in a financial context. Again the total number of distinct network topologies is assumed to be known *a priori *and the network evolution is restricted to changing at most a single edge at a time. More recently (2007-2008) methods in which the number of distinct regulatory phases is determined *a posteriori *have been proposed. Fujita *et al*. [11] developped a Dynamic Vector Autoregressive model to estimate time-varying gene regulatory networks. Notably, only the values of the network parameters change over time, meaning that the global topology of the network remains constant. Xuan and Murphy [12] introduced an iterative procedure based on a similar modelling *ansatz*, which switches between a convex optimization approach for determining a suitable candidate graph structure and a dynamic programming algorithm for calculating the segmentation of the time into distinct phases, *i.e*. the sets of successive time-points for which the graph structure remains unchanged. This time, the number of phases is explicitly determined, but it requires that the graph structure is decomposable. Finally, Robinson and Hartemink [13] used a MCMC sampler for the inference of non-stationary dynamical Bayesian networks, with the attractive feature that the network structure within a temporal phase depends on the structure of the contiguous phases.

The approaches cited so far produce global network topologies with global changes, meaning that all the genes of the network change their regulatory inputs simultaneously. In reality however, we would rather expect that each gene (or at most a subset of genes) has its own and characteristic regulatory pattern. To that end, Rao *et al*. [14] developed a method called *regime-SSM*, which is divided into two steps. The main idea is to first cluster the genes that share the same temporal phases before inferring, in a second step, the network topology describing the regulatory associations between genes within each cluster using an expectation-maximization (EM) algorithm. Ahmed and Xing [15] introduced in 2009 a machine learning algorithm (called TESLA) to infer time evolving networks (that are gene-specific), by solving a set of temporally smoothed *l*_1_-regularized logistic regression problems *via *convex optimization techniques.

The challenge of inferring time-varying structures of gene regulation networks is only starting to be adressed and in this paper we present the ARTIVA algorithm (Auto Regressive TIme VArying models) that is particularly well-suited for addressing the issues raised above. Starting from time-course gene expression data, ARTIVA performs a gene-by-gene analysis and infers simultaneously (*i*) the topology of the regulatory network, and (*ii*) how it changes over time. In order to strike a balance between model refinement and the amount of information available to infer the model parameters, the ARTIVA model delimits temporal segments for each gene where the influence factors and their weights can be assumed homogeneous. For that we use a combination of efficient and robust methods: dynamical Bayesian networks (DBN) to model directed regulatory interactions between genes and Reversible Jump MCMC for inferring simultaneously the times when the network changes and the resulting network topologies. We evaluate the performance of ARTIVA on simulated data and illustrate our approach in the context of two different biological systems. We start by analyzing a commonly used dataset related to the developmental stages of *Drosophila melanogaster *and demonstrate the utility of our approach by a comparative analysis of the ARTIVA results with the TESLA results [15]. Next, we analyze the response of the yeast *Saccharomyces cerevisiae *to benomyl poisoning. This dataset represents an important challenge for the inference of time-varying networks since (*i*) the number of time-points is extremelly small (only 5 time points) and (*ii*) the expression values combine measurements obtained in wild-type and knock-out yeast strains. The biological relevance of the results obtained with ARTIVA are finally assessed using functional annotations and transcription factor binding information.

## Methods

### Graphical models

**Figure 1:**
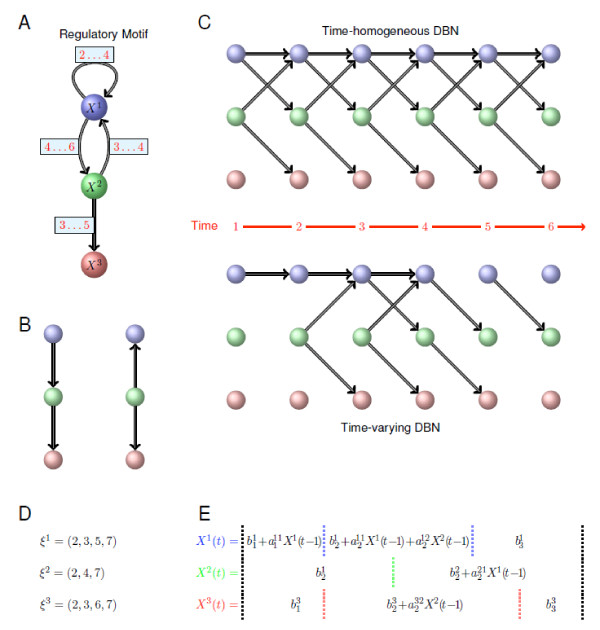
**Illustration of the time-varying DBN formalism**. (A) Regulatory motif among three genes that we wish to model. Crucially, regulatory interactions do not persist over the whole time course considered here, but are turned "on" and "off" at different times. The labels on the edges indicate at what times an edge points to or influences the expression of the target gene.(B) Because Bayesian networks (BNs) are constrained to have a directed acyclic graph (DAG) structure, they cannot contain loops or cycles. Therefore the motif in (A) can only be imperfectly represented using a conventional BN formalism which does not take temporal ordering into account; if *X*^3 ^is statistically independent of *X*^1 ^provided *X*^2 ^is known, we can construct two alternative representations, *P*(*X*^1^, *X*^2^, *X*^3^) = *P*(*X*^3^|*X*^2^).*P*(*X*^2^|*X*^1^).*P*(*X*^1^) and *P*(*X*^1^, *X*^2^, *X*^3^) = *P*(*X*^3^|*X*^2^).*P*(*X*^1^|*X*^2^).*P*(*X*^2^).(C) If time-course expression measurements are available we can unravel the feedback cycles and loops over time. Such dynamical Bayesian networks (DBN) represent the interactions by assuming that at each given time, all the parental nodes come from the previous time point. At the top of this panel we show the DBN constructed assuming a time-homogenous DBN; at the bottom of (C) we show the time-varying DBN constructed by the new algorithm. (D) Changepoint vectors for each of the three genes obtained for the time-varying DBN representation of the motif in (A). (E) The sets of regression models corresponding to the three nodes *X*^1^, *X*^2 ^and *X*^3 ^in the inferred phases. Vertical dotted lines correspond to changepoints separating distinct phases for each node. Compulsory changepoints at the start and the end of the process (*i.e*. at *t *= 2 and *t *= *n *+ 1) are indicated by the black dotted lines; inferred changepoints for each gene are shown in blue, green and red, corresponding to the colours of the genes (as used in parts (A), (B) and (C) of this figure).

Bayesian Networks (BNs) have become a popular framework for representing regulatory networks [6,16] as they offer both a probabilistic interpretation of dependencies among expression of genes and a graphical representation that is more readily accessible than mathematical expressions (see Figure 1). For example if the expression level of gene *i*, here denoted by *X^i^*, determines the expression level of genes *j *and *k*, a diagram such as

j←i→k

can be drawn and the joint distribution of gene expression levels written,

$$P\left(X^{i},X^{j},X^{k}\right)=P\left(X^{k}|X^{i}\right)P\left(X^{j}|X^{i}\right)P\left(X^{i}\right),$$

where *P*(*a*|*b*) denotes the probability of *a *conditional on *b*. Because BNs aim to represent the joint probability distribution (in our case for the expression levels of *p *genes) the corresponding graphical representation is limited to graphs which contain no cycles (Figure 1B). This means that closed loops or complex feed-back structures (as in Figure 1A) cannot faithfully be represented, whereas they are known to pervade regulatory networks [17].

With time-course measurements, this limitation can be overcome by employing a Dynamical Bayesian Network (DBN) formalism [18], where the expression levels of all the genes in a system are modelled as a generally discrete-time stochastic process (Figure 1C). For *p *genes and *n *measurements the expression levels are written as *X^i^*(*t*), with 1 ≤ *i *≤ *p *and 1 ≤ *t *≤ *n*. The joint probability distribution over the expression levels of all genes and at all times is then partitionned, *P*(*X*^1^(1), ..., *X^p^*(1), ..., *X*^1^(*n*), ..., *X^p^*(*n*)), into a product of conditional probabilities of the Markov form:

(1) $$P(X^{i}(t)|X^{r}(t-1),\;...,\;X^{s}(t-1))$$

This means that the expression level of gene *i *at time *t *depends on the expression levels of genes *r*, ..., *s *at time *t *- 1. Genes *r*, ..., *s *are called the 'parents' of gene *i *and denoted by Pa*^i ^*(reciprocally gene *i *is called the 'target' gene of genes *r*, ..., *s*). By making the time dependence of expression levels explicit, loops and feedback interactions can be represented simply by requiring only that the expression of gene *i *at time *t *is independent of all other genes at the same time *t*. In conventional DBN inference approaches it is assumed that the conditional dependencies in Eqn. (1), and hence the set Pa*^i^*, are independent of time *t*. Of course it is possible to allow *X^i^*(*t*) to depend on expression levels *X^r^*(*t *- *τ *) with *τ ***>**1, *i.e*. allow for higher order dependencies. For computational reasons, however, our analysis is restricted to first order Markov processes.

### ARTIVA network model

Let *p *be the number of observed genes and *n *the number of time-points at which expression levels are measured for each gene. In this study, the discrete-time stochastic process *X *= {*X^i^*(*t*); 1 ≤ *i *≤ *p*, 1 ≤ *t *≤ *n*} is considered, taking real values and describing the expression level of the *p *genes at *n *time-points. We start by modelling the gene expression levels at time *t *probabilistically by a vector-autoregressive process:

(2) ∀t≥2, X(t)=A(t)X(t−1)+B(t)+ϵ(t) with ϵ(t)~N(0, Σ(t)),

where ***X***(*t*) = (*X^i^*(*t*))_1≤*i*≤*p *_and N (0, **Σ **(*t*)) is the multivariate normal distribution centered at 0 with diagonal covariance matrix **Σ**(*t*). Note that diagonality of **Σ **ensures that the process describing the temporal evolution of gene expression -- here a first order autoregressive process -- can be represented by a Directed Acyclic Graph (DAG) as in Figure 1C, *i.e*. no edges between nodes at the same time, and where the edges from time *t ***- **1 to time *t *are defined by the set of non zero coefficients in matrix ***A***(*t*) [19]. Furthermore the error in expression measurements of gene *i *does not affect the expression measurements of the other genes and off-diagonal elements in **Σ **can be set to 0.

Crucially, the coefficient matrix ***A***(*t*) = (*a^ij^*(*t*))_1≤*i*, *j*≤*p *_-- which is the adjacency matrix of the gene regulatory network [19,20] -- and the column vector ***B***(*t*) = (*b^i^*(*t*))_1≤*i*≤*p *_-- which is the baseline gene expression that does not depend on the parent gene regulatory controls -- are allowed to vary explicitly with time. This could for example reflect switching on or off of regulatory interactions, *e.g*. in response to developmental, physiological or environmental signals (Figures 1C, D and 1E).

For each gene, *i*, a set of time-points for which the regulatory inputs of the gene change is determined. These time-points are referred to as 'changepoints' and delimit homogeneous phases, *i.e*. sets of time-points for which the local network topology (edges between gene *i *and its parents Pa*^i ^*) remains unchanged. Assuming *k *changepoints, the changepoints are denoted by $\xi^{i}=(\xi_{0}^{i},\;...,\;\xi_{k+1}^{i})$, where $\xi_{0}^{i}=2$ (for a 1*^st ^*order Markov model) and $\xi_{k+1}^{i}=n+1$ (to delimitate the bounds). The distinct phases are labelled by the index of their respective right changepoints. For all times *t *in the phase *h *of gene *i *(*i.e*. $\xi_{h-1}^{i}\leq t<\xi_{h}^{i}$), the *i*-th row of matrix ***A***(*t*) and coefficient *b^i^*(*t*) are assumed constant:

(3) $$\begin{array}{ccc} \forall \xi_{h-1}^{i}\leq t<\xi_{h}^{i}\text{,} & b^{i}(t)=b_{h}^{i}, & \forall 1\leq j\leq p,\;a^{ij}(t)=a_{h}^{ij} \end{array}$$

For phase *h *of gene *i*, the parents ${\text{Pa}}_{h}^{i}$ of gene *i *include every gene *j *such that the coefficient $a_{h}^{ij}$ differs from 0: ${\text{Pa}}_{h}^{i}=\{j;\forall 1\leq j\leq p,\;a_{h}^{ij}\neq 0\}$. Hence for $\xi_{h-1}^{i}\leq t<\xi_{h}^{i}$ the expression of gene *i *is modelled in a regression framework as:

(4) $$\begin{array}{cc} X^{i}(t)=\sum_{j\in {\text{Pa}}_{h}^{i}}a_{h}^{ij}X^{j}(t-1)+b_{h}^{i}+e^{i}(t), & with\;e^{i}(t)\sim N(0,\;{\left(\sigma_{h}^{i}\right)}^{2}), \end{array}$$

where *X^j^*(*t *- 1) is the expression level of gene *j *at time *t *- 1. This defines a multiple changepoint regulatory network, with changepoint positions $\xi ={\left(\xi_{0}^{i},\;...,\;\xi_{k+1}^{i}\right)}_{1\leq i\leq p}$, and the phase-specific regression model parameters, $\left\{a_{h}^{ij},\;b_{h}^{i},\;\sigma_{h}^{i}\right\}$ for all *h*, *i*, *j*. All non-zero coefficients, $a_{h}^{ij}$, indicate relationships between expression levels of genes *i *and *j*, and hence are good indicators of putative biological interactions between those genes.

### Model inference via reversible jump MCMC sampling

#### General principle

We want to infer the autoregressive time-varying network model, which belongs to the overall parameter space that is the union of the parameter spaces of all phases delimited by *k *changepoints (*k *= {0, ..., $\bar{k}$}). Furthermore, for each phase the number of incoming edges on each node (or the network topology) is unknown. Adding or removing a changepoint results in a change in the dimension of the system's state-space: for each additional changepoint a new network topology has to be estimated, and for each deleted changepoint the results previously obtained for the two distinct phases have to be reconciled. Thus, the dimension of the model is unknown and can vary substantially. In order to infer the posterior distribution Pr(*k*, *ξ*, *s*, Pa, *θ*, *σ*|*x*) given the observed data *x *over all of the system's parameters, we used a Reversible Jump Markov Chain Monte Carlo (RJ-MCMC) procedure. The principle of RJ-MCMC lies in constructing a reversible Markov chain sampler that can jump between parameter subspaces of different dimensions; thus allowing the generation of an ergodic Markov chain whose equilibrium distribution is the desired posterior distribution [21,22].

**Figure 2:**
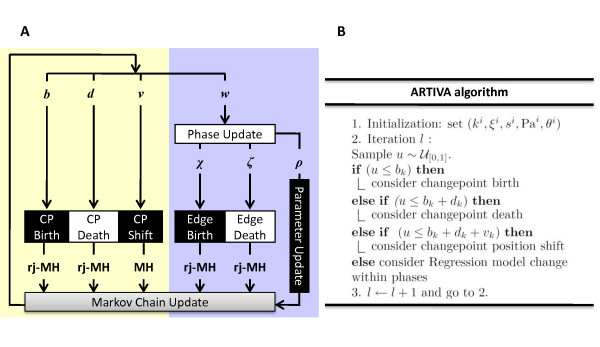
**Illustration of the ARTIVA procedure**. (A) Schematic illustration of the two-step RJ-MCMC scheme used for determining the stationary distribution of time varying dynamic Bayesian networks. With probabilities *b*, *d *and *v*, we propose the birth, death or shift of a changepoint (CP) respectively; with probability *w *we propose an update of the regression model describing regulatory interactions for a gene within a temporal phase. Varying the number of CPs or the number of edges (network topology) corresponds to a change in the dimension of the state-space and is dealt with by using Green's RJ-MCMC formalism [21]. Proposed shifts in changepoint positions are accepted according to a standard Metropolis-Hastings step. Because of conservation of probability we necessarily have *b *+ *d *+ *v *+ *w *= 1 and *χ *+ *ζ *+ *ρ *= 1. (B) Outline of the ARTIVA inference procedure.

Presented in Figure 2, our inference procedure allows us to simultaneously consider all possible combinations of changepoints and network topologies within the different phases. In the RJ-MCMC procedure, the likelihood of the expression measurements *x*(1) observed at time-point *t *= 1 is denoted by Pr(*x*(1)). From the hierarchical structure of the overall parameter space, the joint probability distribution over all parameters can thus be written as the product:

(5) $$\text{Pr }(\;k,\;\xi ,\;s,\;Pa,\;\theta ,\;\sigma ,\;x)\;=\text{Pr}(x(1))\prod_{i=1}^{p}\{\text{Pr}(k^{i},\;\xi^{i},\;s^{i},\;{\text{Pa}}^{i},\;\theta^{i},\;\sigma^{i},\;x^{i})\}.$$

#### Posterior distribution

For each gene, *i*, we construct a RJ-MCMC sampler that directly samples from the joint distribution:

(6) $$\begin{array}{l} \text{Pr}(k^{i},\;\xi^{i},\;s^{i},\;{\text{Pa}}^{i},\;\theta^{i},\;\sigma^{i},\;x^{i})={\text{Pr}}_{\overset{ª}{k}}(k^{i})\text{Pr}(\xi^{i}|k^{i})\\ \;\;\;\;\;\;\;\;\;\;\;\;\;\;\;\;\;\;\;\;\;\;\;\;\;\;\;\;\;\;\;\;\;\;\;\;\;\;\;\;\;\;\;\;\;\;\;\;\;\;\;\;\;\;\;\;\;\;\;\;\;\;\;\;\;\;\;\;\;\;\;\;\;\;\;\;\;\;\;\;\;\;\;\;\;\;\;\;\prod_{h=1}^{k^{i}+1}\text{P}\text{r}(s_{h}^{i},\;{\text{Pa}}_{h}^{i},\;\theta_{h}^{i},\;\sigma_{h}^{i})\text{Pr}(x_{h}^{i}|\xi_{h-1}^{i},\;\xi_{h}^{i},\;s_{h}^{i},\;{\text{Pa}}_{h}^{i},\;\theta_{h}^{i},\;\sigma_{h}^{i}), \end{array}$$

Where ${\text{Pr}}_{\bar{k}}(k^{i})$ Pr(*ξ^i^|k^i^*) and are respectively the prior probabilities of the number of changepoints *k^i ^*and of the changepoint position vector *ξ^i ^*for gene *i*, and where $\text{Pr}(s_{h}^{i},\;{\text{Pa}}_{h}^{i},\;\theta_{h}^{i},\;\sigma_{h}^{i})$ is the prior probability of the parameters defining the regression model of the phase *h *of gene *i*. Finally:

(7) $$\text{Pr}(x_{h}^{i}|\xi_{h-1}^{i},\;\xi_{h}^{i},\;s_{h}^{i},\;{\text{Pa}}_{h}^{i},\;\theta_{h}^{i},\;\sigma_{h}^{i})=\prod_{\xi_{h}^{i}\leq t<\xi_{h+1}^{i}}\text{Pr}(x^{i}(t)|\xi_{h-1}^{i},\;\xi_{h}^{i},\;s_{h}^{i},\;{\text{Pa}}_{h}^{i},\;\theta_{h}^{i},\;\sigma_{h}^{i})$$

is the likelihood of the expression levels $x_{h}^{i}={\left(x^{i}(t)\right)}_{\xi_{h}^{i}\leq t<\xi_{h+1}^{i}}$ of gene *i *observed during phase *h*, and is a realization of the Gaussian distribution defined in Equation(4).

#### Priors

In order to reinforce sparsity of the network and following multiple changepoint approaches involving RJ-MCMC [23,24], we assume the number of changepoints *k^i ^*to be distributed *a priori *as a truncated Poisson random variable with mean *λ *and maximum $\bar{k}$,

(8) $$\forall k^{i}\leq \bar{k},\;{\text{Pr}}_{\bar{k}}(k^{i})\propto e^{-\lambda }\frac{\lambda^{k^{i}}}{k^{i}!}1_{\left\{k^{i}\leq \bar{k}\right\}}.$$

Similarly, the prior probability for the number of parents is a truncated Poisson distribution ${\text{Pr}}_{\overset{ª}{s}}(s_{h}^{i})$ with mean Λ and maximum $\bar{s}$. Here *λ *and Λ can be interpreted as the expected number of changepoints and parent variables, respectively. Following [25], *λ *and Λ are drawn according to a Gamma distribution: λ, Λ~Ga(α, β) where the shape parameter *α *and the scale parameter *β *are chosen so that the prior probability decreases when the numbers of changepoints or parents increase (we set *α *= 1, *β *= 0.5, see Additional file 1 for an illustration of the corresponding distribution). Conditional on there being *k^i ^*changepoints, we assume that the changepoint positions vector *ξ^i ^*takes only non-overlapping and uniformly distributed integer values. The prior for the regression model parameters (*s^i^*, Pa*^i ^*, *θ^i^*, *σ^i^*) are chosen following Andrieu and Doucet' RJ-MCMC procedure for regression model selection [25], based on a work proposed in [26]. The sets of parents Pa*^h^*(*i*) are assumed to be uniformly distributed conditional on $|{\text{Pa}}^{h}(i)|\;=s_{h}^{i}$. The variance, $\sigma_{h}^{i}$, is assumed to be distributed according to a conjugate inverse-Gamma prior distribution with shape parameter *υ*_0_/2 and scale parameter *γ*_0_/2, ${\left(\sigma_{h}^{i}\right)}^{2}\sim ℐG(v_{0}/2,\;\gamma_{0}/2)$. By choosing *υ*_0 _= 1 and *γ*_0 _= 0.1, we set up to Jeffrey's vague prior, $\text{Pr}({\left(\sigma_{h}^{i}\right)}^{2})\propto 1/{\left(\sigma_{h}^{i}\right)}^{2}$[25]. Finally, conditional on $\sigma_{h}^{i}$, the prior distribution for the regression model parameters can be written as,

(9) $$\text{Pr}(s_{h}^{i},\;{\text{Pa}}_{h}^{i},\;\theta_{h}^{i}|\sigma_{h}^{i})={\text{Pr}}_{\overset{ª}{s}}(s_{h}^{i})\text{Pr}({\text{Pa}}_{h}^{i}|s_{h}^{i})\text{Pr}(\theta_{h}^{i}|\sigma_{h}^{i},\;s_{h}^{i},\;{\text{Pa}}_{h}^{i}).$$

Given the parent gene set ${\text{Pa}}_{h}^{i}$ of size $s_{h}^{i}$, the $s_{h}^{i}+1$ regression coefficients, $\theta_{{\text{Pa}}_{h}^{i}}=(b_{h}^{i},\;{\left(a_{h}^{ij}\right)}_{j\in {\text{Pa}}_{h}^{i}})$, are assumed to be drawn from zero-mean Gaussian distributions with covariance ${\left(\sigma_{h}^{i}\right)}^{2}\Sigma_{{\text{Pa}}_{h}^{i}}$,

(10) $$\text{Pr}(\theta_{h}^{i}|\sigma_{h}^{i},\;s_{h}^{i},\;{\text{Pa}}_{h}^{i})=|2\pi {\left(\sigma_{h}^{i}\right)}^{2}\Sigma_{{\text{Pa}}_{h}^{i}}{|}^{-1/2}\exp\left[-\frac{\theta_{{\text{Pa}}_{h}^{i}}^{ˆ}\sum_{{\text{Pa}}_{h}^{i}}^{-1}\theta_{{\text{Pa}}_{h}^{i}}}{2{\left(\sigma_{h}^{i}\right)}^{2}}\right],$$

where the symbol † denotes matrix transposition, $\sum_{{\text{Pa}}_{h}^{i}}=\;\delta^{-2}D_{{\text{Pa}}_{h}^{i}}^{ˆ}(x)D_{{\text{Pa}}_{h}^{i}}(x)$ and $D_{{\text{Pa}}_{h}^{i}}(x)$ is a matrix of size $m^{i}(\xi_{h}^{i}-\xi_{h-1}^{i})\times (s_{h}^{i}+1)$, whose first column is a vector of 1 when the regression model includes a constant, and each *j *+ 1*^th ^*column contains the observed (eventually repeated) value ${\left(x_{tl}^{j}\right)}_{(\xi_{h-1}^{i}-1)\leq t<(\xi_{h}^{i}-1);1\leq l\leq m}$ for all parent gene *j *in ${\text{Pa}}_{h}^{i}$. We did not use shrinkage priors here because the truncated Poisson prior for the number $s_{h}^{i}$ of parents already favours dimension reduction. The term *δ^2 ^*represents the expected signal-to-noise ratio and is sampled according to an Inverse Gamma distribution $\delta^{2}\sim ℐG(\alpha_{\delta^{2}},\;\beta_{\delta^{2}})$ with $\alpha_{\delta^{2}}=2$ and $\beta_{\delta^{2}}=0.2$.

A noticeable advantage of the model is that the marginalization over the regression parameters (*θ^i ^*, *σ^i ^*) in the posterior distribution is analytically tractable,

(11) $$\text{Pr}(k^{i},\;\xi^{i},\;s^{i},\;{\text{Pa}}^{i},\;x^{i})=\int \int \text{Pr}(k^{i},\;\xi^{i},\;s^{i},\;{\text{Pa}}^{i},\;\theta^{i},\;\sigma^{i}|x^{i})d\theta^{i}d\sigma^{i}$$

(see Additional file 1, Section 2 for more details). Then the proposals are sampled from the analytical expression of the network topology posterior distribution (11) -- which is proportional to Pr(*k^i ^*, *ξ^i ^*, *s^i ^*, Pa*^i^*|*x*) -- and the acceptance probability depends on the network topology (*ξ^i ^*, Pa*^i^*) only.

#### Moves

In order to traverse the parameter space of unknown dimension we propose here four different update moves (see Figure 2 and Additional file 1): birth of a new changepoint (*B*); death (removal) of an existing changepoint (*D*); shift of a changepoint to a different time-point (*S*); and update of the regression model defining the network topology within the phases (*R*). These moves occur with probabilities *b_k _*for *B*, *d_k _*for *D*, *v_k _*for *S *and *w_k _*for *R*, depending only on the current number of changepoints *k^i ^*and satisfying *b_k _*+ *d_k _*+ *v_k _*+ *w_k _*= 1. The changepoint birth and death moves represent changes from, respectively, *k^i ^*to *k^i ^*+ 1 phases and *k^i ^*to *k^i ^***- **1 phases. We impose *d*_0 _= *v*_0 _= 0 and $b_{\bar{k}}=0$ to preserve the restriction on the number of changepoints. Otherwise, these probabilities are chosen as follows:

(12) $$b_{k}=c\min\left\{1,\;\frac{{\text{Pr}}_{\overset{ª}{k}}(k^{i}+1)}{{\text{Pr}}_{\overset{ª}{k}}(k^{i})}\right\},\;d_{k+1}=c\min\left\{1,\;\frac{{\text{Pr}}_{\overset{ª}{k}}(k^{i})}{{\text{Pr}}_{\overset{ª}{k}}(k^{i}+1)}\right\}$$

where ${\text{Pr}}_{\bar{k}}$ is the prior distribution for the number of changepoints and the constant *c *is chosen to be smaller than 14 so that the regression model updates and changepoint position shifts are proposed more frequently than births and deaths of changepoints. This improves our ability to infer changepoint positions and the network structures (using the vector-autoregressive framework) within the different phases. Proposed shifts in changepoint positions are accepted using a standard Metropolis-Hastings step, while regression model updates within phases invoke a second RJ-MCMC criterion, which was adapted from the model selection approach of Andrieu and Doucet [25]. As proposals are sampled from the analytical network topology posterior distribution (11), the generation of the regression model parameters $\left(\theta_{h}^{i},\;\sigma_{h}^{i}\right)$ is optional. Together the four moves *B*, *D*, *S *and *R *allow the generation of samples from probability distributions defined on unions of spaces of different dimensions for both the number, *k^i^*, of changepoints and the number $s_{h}^{i}$ of parents within each phase for gene *i*.

### Model selection

Given *a priori *probabilities, the ARTIVA algorithm produces posterior probability estimations over the algorithm iterations for changepoint vectors and network topologies. These posterior probabilities give a detailed picture of all the results and allow in depth analyses of the entire regulatory network architecture. In this study we use in complement to posterior probabilities, the Bayes factor, *i.e*. the ratio of the posterior odds of an hypothesis over its prior odds [27]. The Bayes factor has the advantage to consider the posterior distribution with respect to the priors and to obtain quantitative measurements of the statistical significance of the ARTIVA results which are comparable between different datasets. As an indication, according to Kass and Raftery [27], a proposition is (*i*) *not *supported when it has a Bayes factor below 3, (*ii*) *positively *supported for a Bayes factor between 3 and 20 and (*iii*) *strongly *supported for a Bayes factor over 20. The performance of ARTIVA is evaluated on synthetic and real data (see the following section) by selecting the network structure according to the following procedure. For each gene *i *we first choose the number *k^i ^*of changepoints having the greatest Bayes factor. Then the *k^i ^*changepoint positions having the highest Bayes factors are selected, and for each resulting phase we finally compute the Bayes factor for the possible parent genes and choose the ones with a Bayes factor greater than 3 (see Additional file 1 for a description of the Bayes factor computation).

### Simulation study

In order to evaluate the accuracy of the ARTIVA algorithm to recover changepoints and network topologies correctly, expression data for randomly defined dynamic networks were generated. With respect to the experimental datasets analyzed later (see the following section), two types of expression data were produced. The first type -- referred to Wild-Type (WT) simulations -- match the 'Drosophila life cycle' data. This dataset contains time-series expression data of several genes and the algorithm must find the correlations between unknown parent genes and each target gene. The second type -- referred to as Knock-Out (KO) simulations -- is equivalent to the 'benomyl' dataset. This dataset only contains time-series expression data of target genes in different genetic contexts: wild-type and knock-out mutants for several transcription factors (TFs).

The simulation procedure for a given target gene, also presented in detail in Additional file 2, involves three main steps. First, the structure of the dynamic regulatory network is defined. This consists of randomly setting the number and the localization of changepoints, thereby defining regulatory phases. Then the parent genes and the corresponding coefficients are chosen for each phase. Once the regulatory network is defined, expression data can be generated from this network model. The expression values of the parent genes are first generated randomly (uniformly drawn from [-2, -0.1] ⋃ [0.1, 2]) and subsequently used to calculate the target gene expression according to the autoregressive model presented in Eqn. (4). The whole procedure is repeated (to represent experimental replicates) and noise is added (to represent experimental variability). Because the simulations use the ARTIVA hypothesis concerning the expression associations between parent and target gene expression profiles (autoregressive model), we expect all the results to be correct under ideal conditions (like absence of noise). Therefore, this simulation protocol evaluates the ARTIVA performance and studies the influence of the following parameters:

• the quantity of noise in the data. For all phases *h *of gene *i*, the noise *e^i^*(*t*) is drawn from a Gaussian distribution $N(0,\;{\left(\sigma_{h}^{i}\right)}^{2})$ with standard deviation $\sigma_{h}^{i}(\sigma_{h}^{i}=0.2,0.4,\;...,\;1.8)$,

• the size of the temporal phases (*phasesize *= 1, 2, ..., 5, 12), and

• for WT simulations only, the number of potential parent genes (*Pa*# = 5, 10, 20, 40). This is not necessary for KO simulations because the potential parent genes are obviously restricted to the transcription factors for which KO data is being generated. Regardless of the number of potential parent genes, a maximum of 5 edges from parent genes to a target gene is allowed.

For each parameter value, 200 gene time-series of length = 12 timepoints were randomly generated with 8 and 4 replicates for WT and KO data respectively. Note that KO simulations present less replicates because each replicate already comprises the measurements of the gene expression levels for each knock-out mutant. All other parameters were set to their default values and are specified in Table 1. The ARTIVA algorithm was run on each expression data set and we compared the proposed network model (selected as described in the previous subsection 'Model selection') with the original one. The ability of the algorithm to recover changepoints was evaluated via the Positive Predictive Value (PPV) and the Sensitivity,

**Table 1:** Performance of ARTIVA on simulated data

		WT simulations				KO Simulations			
Parameter		Changepoint sensitivity	Changepoint PPV	Edges sensitivity	Edges PPV	Changepoint sensitivity	Changepoint PPV	Edges sensitivity	Edges PPV
	**0.2**	94.2	95.1	73.9	99.2	100	100	100	98.5
	**0.4**	90.8	94.1	79.3	97.9	100	99	100	98.2
	**0.6**	87.8	92.5	73.9	96.9	96	97.1	99.2	95.4
	**0.8**	75.4	96.3	78.8	96.4	81.4	97.8	97.6	91.4
**Noise**	**1**	80.5	96.6	74.7	97.5	69.1	95	95.1	88.8
	**1.2**	71.7	96.2	78.4	97.6	28.1	82.4	97.2	87.6
	**1.4**	58.7	94.6	79	95.9	23.6	89.7	92.7	87.5
	**1.6**	52.9	91.8	74.4	97.2	10.8	75.9	79.1	86.8
	**1.8**	60.8	94.5	76	95.6	4.8	81.8	76.8	85.6

	**1**	78.5	97.5	1.3	100	79	98.8	18.1	82.4
	**2**	92	92	24.7	98.5	97	96.5	98.7	92.5
**Phase size**	**3**	90	94.2	50.8	98.6	99.5	99	100	93.6
	**4**	94.5	94	74.5	98.6	99.5	99.5	100	96.4
	**5**	96	99	76.9	96.8	99.5	97.5	100	94.7
	**12**	100	99	92.6	98	99	97.1	100	97.8

	**5**	93.7	95.5	82.4	99.1	_	_	_	_
**# of parent**	**10**	81.1	88.3	69.7	96.4	_	_	_	_
**genes**	**20**	62.4	83.4	51.2	97.3	_	_	_	_
	**40**	54	77.2	33.1	96.4	_	_	_	_

To evaluate ARTIVA performances, two types of data were used: WT simulations corresponding to time-series expression data with no knowledge of potential transcription factors and KO simulations corresponding to several time-series expression data of a simulated *wild-type *strain and *knock-out *strains for each known transcription factor (see Methods and Additional file 2 for more details). The default values of the parameters used for the simulation study are: # of timepoints *n *= 12; # of changepoints k~U({1, .., n/4}); maximal # of edges = 5; parent to target coefficient ~U([−2, −0.1]∪[0.1,2]); phase sizes ~U({3, .., n});# # of replicates *r *= 8 for WT simulations, *r *= 4 for KO simulations; noise standard deviation ~N(0,0.5); total # of simulations = 200 for each condition. In this table, the value of noise intensity, phase size and number of parent genes change according to the parameter under study (all other parameters were set to default). In each condition, the ability of ARTIVA to detect *all *true phase changepoints and model edges (Sensitivity) and to detect *only *true positives (Positive Predictive Value, PPV) was calculated. Overall, this simulation study allows us to gain confidence in ARTIVA results (≃ 80% of PPV and sensitivity) for a given set of parameters (noise standard error is on the order of the mean value of the regression coefficients, number of measurements in a regulatory phase > 8 and less than 20 parent genes).

(13) $$PPV=\frac{TP}{\left(TP+FP\right)}\;Sensitivity\;=\frac{TP}{\left(TP+FN\right)}$$

with TP = True Positives, FP = False Positives and FN = False Negatives. The edges PPV and Sensitivity was computed for the phases whose changepoints were correctly inferred.

### Microarray data

The first microarray dataset -- referred to as '*Drosophila *life cycle' data -- was produced by Arbeitman *et al*. [28]. It includes the mRNA expression levels of 4028 genes at 67 successive time-points spanning the four stages of the *D. melanogaster *life cycle: the embryonic (31 time-points), larval (10 time-points) and pupal stage (18 time-points) and the first 30 days of adulthood (8 time-points). Expression data were collected from the Gene Expression Omnibus database: http://www.ncbi.nlm.nih.gov/geo/. 4005 genes with consistent annotation are used for the analysis. Potential parent genes were restricted to genes with known transcriptional activity based on Gene Ontology information [29]. Hence, 136 genes were selected as potential parents. They belong to one of the four following Gene Ontology molecular functions: 'Transcription activator activity' (GO:0016563), 'Transcription repressor activity' (GO:0016564), 'Transcription factor activity' (GO:0003700) and 'Transcription cofactor activity' (GO:0003712). For each target gene, we gave priority to the 10 potential parent genes with the most highly correlated gene expression profiles over any successive 10 time-points.

The second microarray dataset -- referred to as 'benomyl' data -- was published by Lucau-Danila *et al*. [30]. In this study, the authors measured the changes in mRNA concentrations for each gene at successive times after addition of benomyl (an antimitotic drug) in the growth media of *Saccharomyces cerevisiae *cells. Parallel experiments were conducted in different genetic contexts: the wild type strain and knock-out (KO) strains in which the genes coding for different transcription factors connected to drug response, *YAP1*, *PDR1*, *PDR3*, and *YRR1*, were deleted. For each yeast strain, the measured expression values for 5 time-points (at 30 s, 2 min, 4 min, 10 min, 20 min) were obtained from the website: http://www.biologie.ens.fr/lgmgml/publication/benomyl. We only considered genes that (*i*) showed significant changes in mRNA levels during the time-course analysis in the WT strain (119 genes presented by Lucau-Danila *et al*. [30]), and (*ii*) had less than 20% of missing expression measurement data in the four KO strains. The resulting expression table comprised data for 78 genes (see Additional file 3 for complete list of genes). Hierarchical clustering was performed applying the 'hclust' function available in the R programming langage http://cran.r-project.org/, using Euclidean distance between gene expression profiles and the 'ward' method for gene agglomeration (see also Additional file 4).

### Technical information

The ARTIVA algorithm is implemented in R programming language. The source code is freely distributed to academic users upon request to the authors. A 50,000 iterations procedure lasts around 5 min times the number of genes for the analysis of 100 time-course measurements (for example 5 replicates over 20 time-points) with a 2.66 GHz Intel(R) Xeon(R) CPU and 4 G RAM.

## Results

### Evaluation of the algorithm performances

To evaluate the performance of our ARTIVA approach, simulations are run in order to assess the impact of three major factors on the algorithm performances: noise in the data, minimal length of phases, and number of proposed parent genes (the latter for WT simulations only, see Methods). Sensitivity and Positive Predictive Value (PPV) calculated for the detection of changepoints and of models, *i.e*. the topology of the network within the phases, are presented in Table 1. In WT simulations, the changepoint sensitivity is greater than 80% when the noise standard error reaches *σ_i _*= 1. As noise increases further, the ability of the algorithm to recover changepoints decreases in terms of sensitivity, but still, the changepoint sensitivity remains greater than 70% when the noise standard deviation reaches *σ_i _*= 1.2 (a value that is larger than the mean value of the regression coefficients, uniformly sampled from [-2; -0.1] ⋃ [0.1; 2]). The WT data was generated with *r *= 8 repeated measurements for each time point, whereas the KO data were simulated with only 4 repeated measurements for each time point (because each measurement includes data from different genetic contexts, see Methods). That is the reason why the changepoint sensitivity with KO simulations starts to decrease with smaller noise standard deviation compared to WT simulations. Nevertheless, the changepoint sensitivity is still greater than 80% even when noise reaches *σ_i _*= 0.8. The number of measurements for each phase also plays an important role for the changepoint detection sensitivity. Indeed, during a phase reduced to a single timepoint, there are only *r *repeated measurements to estimate the autoregressive models. Interestingly, the ARTIVA algorithm here succeeds in finding the correct dynamic networks with a sensitivity value of 79% for a phase size of 1, in both WT and KO simulations (default noise standard error *σ_i _*= 0.5). With phases of size 2, the changepoint sensitivity is greater than 90%. For all noise levels considered here the changepoint PPV is greater than 95%; furthermore changepoint PPV appears to be stable and not to be affected by the phase size either. Knock-out data are usually collected for a restricted number of knock-out genes and the number of possible parents is limited. However, wild-type experiments give expression time series data for a large number of genes at once. The number of proposed parents increases the dimension of the model and the estimation procedure accuracy is expected to be affected as the dimension increases. Here, the changepoint sensitivity obtained with ARTIVA is still 54% when the parent genes are chosen from among a set of 40 proposed parents. The changepoint sensitivity goes up to 81% when the number of potential parents is reduced to 10. The changepoint PPV is only slightly affected by the number of proposed parents. The PPV is still greater than 75% when the number of potential parents is 40.

The edge detection in Table 1 was evaluated when the correct changepoint segmentation was recovered. Once the correct changepoints are recovered, neither noise nor short phases appear to strongly affect the detection of edges. The edge sensitivity deteriorates for extreme situations only. Indeed, the edge sensitivity is equal to 18% when phase size is 1 for KO simulations. For WT simulations, the edge sensitivity is about 50% when phase size is 3 or when the number of proposed parents is 20. In all other cases, the edge sensitivity is greater than 75% and the edge PPV is greater than 95%.

Simulation studies such as the one performed here do, of course, only provide a partial insights into an algorithms performance and robustness. They are nevertheless essential to gain confidence in the performance of novel algorithms and to develop understanding of their likely limitations. Together these results serve to illustrate of the robustness of the ARTIVA algorithm. In particular, ARTIVA can deal with some of generic problems encountered in real experimental data. It still performs well when noise standard error is on the order of the mean value of the regression coefficients, when the number of measurement per phase is reduced to 8 or when the number of possible parents reaches 20. At some point, the ARTIVA algorithm misses some changepoints, but the PPV is still very large, meaning that we can have great confidence in the changepoints having a high posterior probability.

### Temporal variation of the Drosophila development transcriptional program

**Figure 3:**
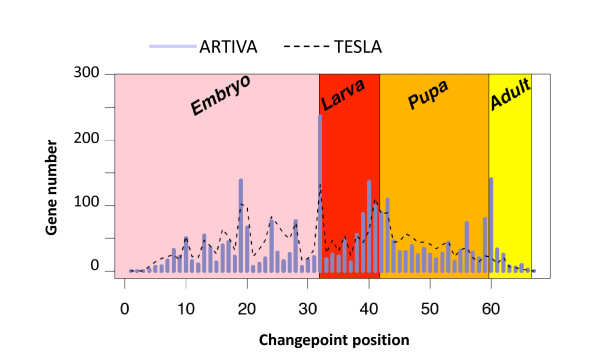
**Changepoints of gene regulation networks across the Drosophila melanogaster development**. Microarray results for the time courses of *Drosophila *life cycle [28] were analysed using ARTIVA. The number of identified changepoints using ARTIVA are shown in blue for each of the 67 time-points. They are compared with the most significant changes identified with the TESLA algorithm [15], shown in black dashed line. Time-intervals for each developmental stage are represented with the following color-code: pink = Embryo (31 time-points), red = Larva (10 time-points), orange = Pupa (17 time-points), yellow = Adult (8 time-points).

In light of the simulation analysis, we then apply our method to the well-studied expression datasets produced by Arbeitman *et al*. [28]. In this study, the authors report gene expression patterns for nearly one-third of all *D. melanogaster *genes during a complete time-course of development. The ARTIVA algorithm is run for each gene for 50,000 iterations, looking for parental relationships with the 10 transcription factors for which gene expression profiles were most highly correlated over any successive 10 time-points (see Methods). Out of the 4005 analyzed genes, 1704 (42%) were found to be involved in the time-varying networks spanning the whole *Drosophila *life-cycle (134 were identified as parent genes, 1623 as target genes and 53 were both parent and target genes). Interestingly, 2583 changepoints were also identified. The distribution over the time-points and with respect to the developmental stages is shown Figure 3. We observe that time intervals {18 to 19}, {31 to 33}, {41 to 43} and {59 to 61} contain more than 40% of the changepoints. Notably the intervals {31 to 33}, {41 to 43} and {59 to 61} include the developmental stage transitions from embryo to larva, from larva to pupa and from pupa to adult, respectively. The high number of changepoints at mid-embryogenesis (interval {18 to 19}) corresponds to a major morphological change related to a modification of transcriptional regulations, as described in [28].

To further evaluate ARTIVA, we compared our results with those obtained using the TESLA algorithm [15]. TESLA has been recently published (2009) and to our knowledge it is with ARTIVA, the only other procedure which recovers time varying regulatory networks where the changepoints are gene specific. As described in [15], we first discretized the expression measurements into two levels: 1 for up-regulation and 0 for down-regulation. The TESLA procedure requires specification of two parameters, *λ*_1_, which is a sparsity coefficient, and *λ*_2_, which is a smoothness penalty coefficient. Several combinations of (*λ*_1_, *λ*_2_) parameters were tested (data not shown), and we finally retained the average values presented by the authors in their simulation study [15], *i.e*. *λ*_1 _= 0.01, *λ*_2 _= 1. The TESLA analysis was run using the same subset of *Drosophila *genes used with ARTIVA, and the 2583 most significant temporal changes identified with TESLA are compared to the 2583 ARTIVA changepoints (Figure 3, dashed line). In agreement with the ARTIVA results, an important number of regulatory changes (28%) occurred during the developmental stage transitions (mid-embryogenesis, embryo to larva, larva to pupa and pupa to adult), but notably this number is significantly lower than the one obtained with ARTIVA (40%, see previous paragraph). This is especially remarkable considering the last phase transition from pupa to adult. The observation of a significant number of changepoints at developmental stage transitions lends credibility and supports our ARTIVA results. Our method appears powerful in inferring the timepoints at which transcriptional control of individual genes switches.

### Time-varying regulatory network involved in the response of Saccharomyces cerevisiae to benomyl poisoning

**Figure 4:**
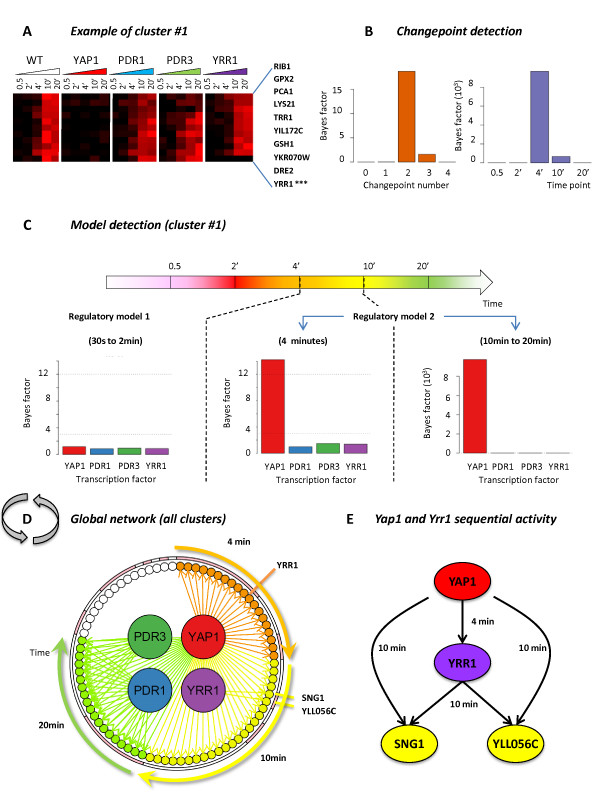
**Time-dependent regulatory network involved in yeast chemical stress response**. Microarray results for the kinetics of benomyl action [30] were analyzed using ARTIVA. A hierarchical clustering analysis was carried out for the time-course responses of the wild-type strain and deletion strains for four transcription factors (TFs): Yap1, Pdr1, Pdr3 and Yrr1. The resulting 18 clusters have low intra-cluster variability and comprise genes whose expression is identically modified in TFs deletion strains compared to the wild-type strain (see Methods). Results for cluster #1 are presented here. (A) Gene expression measurements represented using the common color code (black for expression values around 0 and red for positive values). Bayes factors for changepoint (CP) and edge detection are respectively shown in (B) and (C). Two CP were identified at the 4 min and 10 min time-points, and regulatory associations with the TF Yap1 were identified in the second temporal phase (from 4 min to 20 min). (D) All the identified regulatory associations are shown here, after analyzing the 18 clusters of co-expressed genes independently. They are all positive, meaning that each transcription factor activates the expression of their respectives target genes. Regulatory interactions are color-coded according to their starting time-point: orange = 4 minutes, yellow = 10 minutes and green = 20 minutes. We found 62 regulatory interactions for Yap1; 19 for Pdr1; 10 for Pdr3; and 2 for Yrr1. Pink and white segments on the surrounding circle indicate genes belonging to the same gene expression cluster, the clusters are ordered as follows (starting at 4 min): 1, 18, 7, 9, 13, 8, 17, 2, 5, 6, 3, 11, 4, 10, 12, 14, 15, 16.

In our second example, we apply ARTIVA to a selected set of 78 gene expression profiles from *Saccharomyces cerevisiae *cells grown under benomyl-induced stress conditions [30] (see Methods). A hierarchical cluster analysis identifies 18 clusters of genes with concordant transcription profiles (see Additional file 5). For each cluster, time varying networks are inferred using the included gene expressions measured in the wild type and four deletion strains (Yap1, Pdr1, Pdr3 and Yrr1), running the RJ-MCMC scheme for 50,000 iterations. Regulatory associations between parent and target genes are proposed if the deletion of a parent gene significantly alters the expression measurements of its target genes (compared to the WT situation) (see Methods). The results are presented in Figure 4 and Dataset S1. As an illustration, the cluster #1 comprises 10 genes (Figure 4A) for which two changepoints are detected at the 4 and 10 minute time-points (Figure 4B), when these genes fall under the regulatory control of Yap1 (Figure 4C). Even if Yap1 is the only transcription factor identified here, its regulatory interactions with the target genes in the third phase are highly significant (Bayes factor = 9.10^3^) compared to those in the second phase (Bayes factor = 14.22). This explains the detection of two changepoints. The results obtained for all other clusters are combined to obtain a global view of the time-varying regulatory network involved in benomyl stress response (Figure 4D). In agreement with the pioneering study of Lucau-Danila *et al*. [30], the transcription factor Yap1 appears to have the predominant role in the benomyl stress response as ARTIVA identified edges with 79% of the analyzed genes (62 associations with clusters # {1, 2, 5, 6, 7, 8, 9, 13, 18, 17}). Also PDR1, being the parent gene of 24% of the genes, exerts significant control (19 associations with clusters # {5, 6}). Pdr3 and Yrr1 present only a small number of target genes (10 associations with cluster #6 and 2 associations with cluster #13, respectively).

Furthermore, our ARTIVA model provides a dynamic classification of the benomyl responsive-genes, based on their time of induction. Such a dynamical point of view can elucidate the chronology of events, especially regarding the Yap1 activity. ARTIVA identified three classes of Yap1 targets, depending on their time of induction: 4 minutes (clusters # {1, 7, 18}, orange arrows Figure 4D); 10 minutes (clusters # {2, 8, 9, 13, 17}, yellow arrows Figure 4D); and 20 minutes (cluster # {5, 6}, green arrows Figure 4D). Almost all the genes included in the earliest group are known to be transcriptionally controlled by Yap1 (95% based on YEASTRACT information [31]). They encode proteins involved in redox control (*GPX2*, *TRR1*, *GSH1*, *GTT2*) or vacuolar transporters (*YCF1*). The middle group contains also an important rate of Yap1 targets (87%), which act at the level of the plasma membrane (*FLR1 *and *FRM2*) or encode proteins involved in response to toxins (for instance *AAD6*, *AAD16*, *ECM4*). Yap1 activity in the last group is partially overlapping with the actions of Pdr1 and Pdr3. Most of the genes in this group have unknown functions, but some of them are still labelled in the YEASTRACT database as being targets for Yap1 (74%), Pdr1 (32%) and Pdr3 (20%). Finally, *YRR1 *deserves a special mention. Unlike the genes that encode the transcription factors Yap1, Pdr1 and Pdr3, the *YRR1 *gene is transcriptionnally activated during the benomyl response. As a consequence, ARTIVA identified *YRR1 *(*i*) as a Yap1 target whose expression was induced 4 minutes after benomyl addition in the cell growth culture (see *** Figure 4A); and (*ii*) as a parent for genes *SNG1 *and *YLL056C *at 10 minutes. Interestingly these observations highlight a sequential activity of Yap1 and Yrr1 transcription factors together with an overlap of their targets (Figure 4E). This regulatory model, in which Yrr1 seconds Yap1, is fully supported by recent experimental data [32].

## Discussion

### ARTIVA: a new statistical modelling framework to learn temporally varying gene-regulation networks

The ARTIVA approach allows us to reverse engineer the temporally varying structure of transcriptional networks by inferring simultaneously the times at which regulatory inputs of genes change and the nature of these incoming inputs. Our approach is computationally efficient and can exploit powerful search heuristics to scan the space of potential incoming edges. Compared to others methodologies recently proposed in the literature, ARTIVA has the major advantage of combining efficient and well-tried techniques (Bayesian networks and RJ-MCMC sampler) in order to solve several related problems. First, with ARTIVA there is no need for prior information regarding either the number of regulatory phases or the number of regulatory interactions between parent and target genes. Starting from uninformative priors (such as truncated Poisson or uniform distributions, see Methods), the posterior distribution for the number of changepoints, their positions and the regulatory models within each recovered phase is directly obtained from the ARTIVA runs. Also, ARTIVA allows the detection of regulatory phases for individual genes. Finally, whereas many approaches -- like Bayesian Dirichlet Equivalent (BDE) score in a dynamic context [13] or the TESLA framework [15]-- require the expression measurements to be discretized, the ARTIVA procedure has the advantage to work directly with continuous datasets. Thus there is no need to set arbitrary thresholds to define up- and down-regulated groups of genes.

We demonstrate the performance of the ARTIVA algorithm by (*i*) applying it to simulated data (Table 1) and (*ii*) performing a comparative analysis of the ARTIVA and TESLA [15] results (Figure 3). Because the simulations were such that they mirror the biological data analyzed afterwards as much as possible, we gain considerable confidence in the output of the ARTIVA approach when used on the two datasets considered here. Overall, the algorithm shows very good performance in retrieving the simulated dynamic networks, except in extremely unfavourable conditions, such as too much noise in the data or time series that are not sufficiently long and dense. These exploratory studies allow us to interpret the ARTIVA outputs more reliably.

### New biological insights into the Drosophila development and the yeast stress response

The two biological networks presented in this study (Figures 3 and 4) are very different, both from a biological and a technical point of view. Their respective analyses represent different challenges for the application of the ARTIVA algorithm. The '*Drosophila *life cycle' data is representative of data used for classical regulatory network inference; successive gene expression measurements spanning a given biological process - here the *Drosophila *development - in order to detect potential regulatory interactions from gene expression profiles. This data is particularly suited for the inference of a temporally varying regulation network, since (*i*) the number of time-points is large (more than 80% of all published time series expression datasets are short with 8 time-points or fewer [33]) and (*ii*) the transitions between the distinct stages of *Drosophila *development {Embryo (E), Larva (L), Pupa (P), Adult (A)} are well-described in the literature [28]. We can thus reasonably expect to identify changepoints precisely at transitions between life stages (Figure 3). On the other hand, discrimination between parent and target genes represents an important additional step towards a complete description of the genetic networks that control development. These inferred temporal changes can form hypotheses as to how we can interfere rationally with developmental processes; *e.g*. arresting development in a given state by selectively knocking down transcription factors or targets at a given developmental stage.

The 'benomyl' dataset represents a particular challenge for ARTIVA to retrieve a dynamic regulatory network for two reasons. First, the number of time-points is extremely small (only 5 time-points), and no replicate data points are available. To manage the lack of data, we cluster genes with concordant transcription profiles and analyze them jointly with ARTIVA. This cluster analysis was possible because the maximal intracluster variability did not exceed 0.2 (see Additional file 4), a value that ARTIVA is able to manage based on our simulation results (Table 1). Second, in this *S. cerevisiae *dataset, it is known that the genes coding for key regulators of the stress response system, *i.e*. transcription factors Yap1, Pdr1 and Pdr3, exhibit flat expression patterns during stress condition (Additional file 4); this prevents the use of correlation measures with their expression profiles to identify causal relationships with their potential target genes. In this context, we needed to adapt the ARTIVA inference procedure in order to integrate gene expression profiles measured in the wild type and KO strains. Regulatory associations between parent and target genes are thus proposed if the deletion of a parent gene significantly alters the expression measurements of its target genes (compared to the WT situation). Compared to the previous study of Lucau-Danila *et al*. [30], the main benefit of ARTIVA analyses is that it provides a dynamic classification of the benomyl response genes (Figure 4). It also points out contributions of the Yrr1 and Pdr3 transcription factors, which were ignored in previous analyses. Interestingly, the versatile and non exclusive joint action of Pdr1 and Pdr3 in chemical stress response, together with the overlap with Yap1 activity, is supported by recent experimental data available on these two factors [32,34,35].

## Conclusions

The comprehensive analysis suggests that the ARTIVA approach allows us to describe and reverse-engineer the dynamic aspects of molecular networks. Such time-varying networks provide a middle ground between networks homogeneous in time and explicit dynamical models. The latter require substantial further information in order to model the dynamics of biological systems [36]. Inferring such systems is a considerable statistical challenge and it has recently been shown that some parameters cannot be inferred with any degree of certainty from time-course data. This so-called sloppy behaviour [37,38] has been identified even in very simple dynamical systems. In contrast to classical network reverse engineering approaches such as dynamic Bayesian networks [18] and graphical Gaussian models [39], ARTIVA also allows us to construct more complex hypotheses where interactions may depend on time.

As no particular constraint is imposed to the changepoint positions or to the succession in network topologies within phases, the ARTIVA model appears to be highly flexible. The results are not *a priori *directed toward any particular regulatory associations between genes. This flexibility can be extremely valuable, especially when no information regarding the studied biological process is available. But the rapid accumulation of data obtained with different experimental approaches gives the opportunity to acquire a more comprehensive picture of all the interactions between cellular components. To understand the biology of the studied systems better, the trend is clearly towards the aggregation of multiple sources of information. A natural future direction in the development of ARTIVA will be to incorporate data originating from different sources in the model. In particular, protein/DNA interaction data (ChIP-chip or ChIP-seq experiments) could be effective by replacing the uniform prior for the edges with a prior favouring edges that correspond to the experimentally identified interactions (see [40,41] for an illustration). Also, ARTIVA assumes independent network topologies within successive phases and can identify very different regulatory associations between two phases, even if the time delay between the phases is very short. This assumption was appropriate in case of biological models like the *Drosophila *development and the yeast stress response, mainly because those are processes in which transcriptional regulations are highly dynamic. However, when considering systems that evolve more smoothly or in case of datasets with a small number of time points, it would be interesting to incorporate a regularization scheme into ARTIVA in order to favour slight changes from one phase to the next one. Such an approach has already been initiated in [13] for discretized data and in [42] where the regularization scheme is based on a common network structure. There are still huge gaps in our knowledge of biological networks and of the dynamics they mediate. What triggers whether or not an interaction is present depends subtly on the cellular context, the complement of molecules inside a cell (if we focus attention of intra-cellular processes and networks) and their respective molecular interactions. Understanding all of these factors and their interplay will ultimately be crucial in order to design biological interventions rationally. But statistically inferring them poses a set of formidable challenges. The use of relatively simple mathematical models (such as vector-autoregressive processes) allows us to distil the essential dynamics of complex temporal processes in biological systems. Thus ARTIVA provides a platform for the analysis of transcriptomic data, which could be straightforwardly expanded to include other data, *e.g*. transcription factor activities or other proteomic measurements.

## Authors' contributions

SL conceived, implemented the first version of ARTIVA algorithm and drafted the manuscript. JB optimized the ARTIVA source code and performed simulations. FD provided help with data analysis. MPHS and GL contributed equally to this work. MPHS provided help with the model selection formalism and drafted the manuscript. GL designed the experiments, performed data analysis and drafted the manuscript. All the authors read and approved the final manuscript.

## Supplementary Material

Additional file 1 **Supplementary Text S1 - Priors illustration and complete mathematical description of the RJMCMC procedure and of the Bayes factor computation**. Click here for file

Additional file 2 **Supplementary Figure S1 - Principle of the simulation study**. Click here for file

Additional file 3 **Supplementary Dataset S1 - Full edge list of the inferred time varying networks of the 'benomyl' data**. Click here for file

Additional file 4 **Supplementary Text S2 - Supplementary results related to the 'benomyl' analyses**. Click here for file

Additional file 5 **Supplementary Figure S2 - Expression measurements for the 18 clusters used in the 'benomyl' analyses**. Click here for file
