# Supplementary material for: Statistical inference of the time-varying structure of gene-regulation networks
Source: BMC Syst Biol. 2010 Sep 22;4:130. doi: 10.1186/1752-0509-4-130 (PMC2955603; doi:10.1186/1752-0509-4-130)
Supplement: Additional file 1 — Supplementary Text S1 - Priors illustration and complete mathematical description of the RJMCMC procedure and of the Bayes factor computation. [file 1752-0509-4-130-S1.PDF]

Text S1 - Additional file for the paper entitled ‘Statistical inference of the time-varying structure of gene-regulation networks’

Sophie Lèbre, Jennifer Becq, Frédéric Devaux, Michael P.H. Stumpf, Gaëlle Lelandais.

Contents

1 Prior distributions for the number of changepoints and the number of parents **2**

2 Move acceptation based on the network structure only **3**

3 Details of the ARTIVA algorithm **4**
3.1 Birth of a changepoint 4
3.2 Death of a changepoint 5
3.3 Shift of a changepoint position 6
3.4 Phase Update (Updating the network topology within phases) 6
3.5 Updating hyperparameters 7

4 Convergence of the algorithm **8**

5 Bayes factor (BF) computation **9**
5.1 BF for the number of changepoints 9
5.2 BF for the changepoints position 10
5.3 BF for the edges (conditional on a chosen phase of the network) 11

1 Prior distributions for the number of changepoints and the number of parents

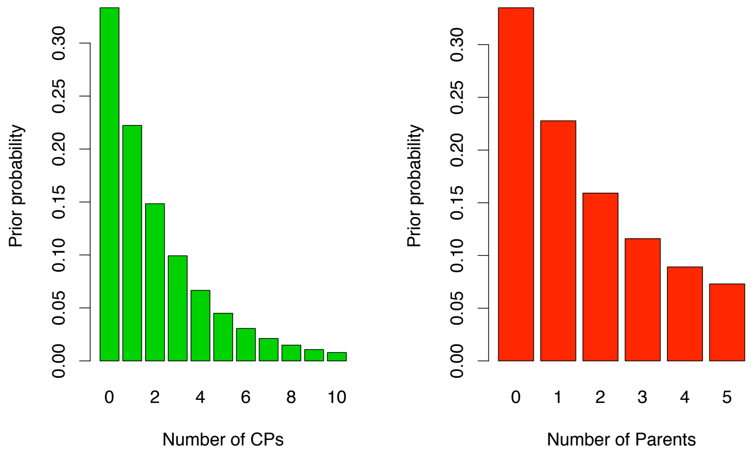

Figure 1: Prior distributions for the number of CPs (left) and the number of parents (right). The number of CPs (k) (respectively the number of parents (s)) is sampled from a truncated Poisson with mean λ (resp. Λ), which is drawn from an Inverse Gamma distribution : $\lambda, \Lambda \sim \mathcal{G}a(\alpha, \beta)$. Here we set the maximal number of CPs $\overline{k} = 10$ and the maximal number of Parents $\overline{s} = 5$. We choose the hyperparameters $\alpha = 1$ and $\beta = 0.5$ in order to limit the dimension and to preserve the network sparsity.

2 Move acceptation based on the network structure only

Following Andrieu and Doucet in their RJ-MCMC approach for model selection [1], we integrate out the joint posterior distribution for parameters $(k^i, \xi^i, s^i, \mathrm{Pa}^i, \theta^i, \sigma^i, x^i)$,

$$\Pr(k^i, \xi^i, s^i, \mathrm{Pa}^i, \theta^i, \sigma^i, x^i) = \Pr(k^i)\ \Pr(\xi^i|k^i) \times \prod_{h=1}^{k^i} \Pr(x_h^i|\xi_{h-1}^i, \xi_h^i, s_h^i, \mathrm{Pa}_h^i, \theta_h^i, \sigma_h^i)\ \Pr(s_h^i, \mathrm{Pa}_h^i, \theta_h^i|\sigma_h^i)\ \Pr(\sigma_h^i) \quad (1)$$

over the parameters (θ^i, σ^i) to obtain an expression of the posterior density $\Pr(k^i, \xi^i, s^i, \mathrm{Pa}^i, x^i)$. The regression model parameters (θ^i, σ^i) are not related to the network topology which is our main interest here. The integration over θ_h^i (normal distribution) and over σ_h^i (inverse gamma distribution) yields,

$$\Pr(k^i, \xi^i, s^i, \mathrm{Pa}^i, x^i) = \int\int \Pr(k^i, \xi^i, s^i, \mathrm{Pa}^i, \theta^i, \sigma^i, x^i) d\theta^i d\sigma^i$$

$$= \frac{\exp(-\lambda)}{norm_\lambda} \lambda^{k^i} \frac{(n-2-k^i)!}{(n-2)!} \left(\frac{\exp(-\Lambda)}{norm_\Lambda} \frac{\left(\frac{\gamma_0}{2}\right)^{\upsilon_0/2}}{\Gamma(\frac{\upsilon_0}{2})} \right)^{k^i+1}$$

$$\prod_{h=1}^{k^i+1} \left\{ \frac{(p-s_h^i)!\ \Lambda^{s_h^i}}{p!\ (\delta^2+1)^{(s_h^i+1)/2}}\ \Gamma\left(\frac{\upsilon_0 + m^i(\xi_h^i - \xi_{h-1}^i)}{2} \right) \left(\frac{\gamma_0 + (x_h^i)^\dagger P_h^i x_h^i}{2} \right)^{-\frac{\upsilon_0 + m^i(\xi_h^i - \xi_{h-1}^i)}{2}} \right\} \quad (2)$$

where $norm_\lambda$ and $norm_\Lambda$ are the normalization constants required by the truncation of the Poisson distributions with mean λ and Λ respectively, and matrices P_h^i, M_h^i and vector d_h^i are defined as follows, with I referring to the identity matrix of size $m^i(\xi_h^i - \xi_{h-1}^i)$,

$$P_h^i = I - D(x_{\tau_h^i}) M_h^i D^\dagger(x_{\tau_h^i}), \quad (3)$$

$$M_h^i = \frac{\delta^2}{\delta^2+1} \left(D^\dagger(x_{\tau_h^i}) D(x_{\tau_h^i}) \right)^{-1}, \quad (4)$$

$$d_h^i = M_h^i D^\dagger(x_{\tau_h^i}) y_h^i \quad (5)$$

where the symbol $\dagger$ denotes matrix transposition, $\Sigma_{\mathrm{Pa}_h^i} = \delta^{-2} D^\dagger_{\mathrm{Pa}_h^i}(x) D_{\mathrm{Pa}_h^i}(x)$ and $D_{\mathrm{Pa}_h^i}(x)$ is a matrix of size $m^i(\xi_h^i - \xi_{h-1}^i) \times (s_h^i + 1)$ whose first column is a vector of 1s when the regression model includes a constant and the $j+1^{th}$ column contains the observed (eventually repeated) value $(x_{tl}^j)_{(\xi_{h-1}^i - 1) \le t < (\xi_h^i - 1); 1 \le l \le m}$ for all parent gene j in Pa_h^i.

We use this posterior distribution for the proposals related to the changepoint structure (CP birth or CP death). The generation of the regression model parameters $(\theta_h^\star, \sigma_h^\star)$ is only optional, and only used when an estimation of their posterior distribution is wished for. Also, a changepoint birth or death acceptance is performed without generating the regression model parameters for the modified phase. Thus the acceptance probability of the move does not depend on the regression model parameters (θ_h, σ_h) but only on the network topology in the phases delimited by the changepoint involved in the move.

3 Details of the ARTIVA algorithm

The technical details of the implementation of ARTIVA herafter. The RJ-MCMC acceptance probability for a changepoint 'birth' can be written as $\min(1, r_{k,k+1})$ where

$$r_{k,k+1} = (\text{posterior distribution ratio}) \times (\text{proposal ratio}) \times (\text{Jacobian}). \tag{6}$$

The changepoint 'death' is accepted with probability $\min(1, r_{k,k-1})$. We outline below the computation of the proposal ratio and the Jacobian in (6). The four different moves are defined using heuristic considerations; our only consideration is that the correct invariant distribution of the Markov chain is maintained. As pointed out in [2], a particular choice of move proposal will only influence the convergence rate of the algorithm but not the properties of the stationary distribution.

3.1 Birth of a changepoint

Let ξ^i be the current changepoint vector containing k^i changepoints. When considering the birth of a new changepoint, we first draw a new changepoint position $\xi^\star$ uniformly among those that do not currently contain changepoints,

$$\xi^\star|\xi^i \ \sim \ \mathcal{U}_{\{3,\ldots,n\}\setminus\{\xi^i\}}. \tag{7}$$

The new changepoint is within current phase $h^\star$ of the target gene i, *i.e.* $\xi^i_{h^\star-1} < \xi^\star < \xi^i_{h^\star}$. This phase starts at changepoint $\xi^i_{h^\star-1}$ and ends at $\xi^i_{h^\star} - 1$, where $\xi^i_0 = 2$ and $\xi^i_{k^i+1} = n+1$ as previously defined. The proposed new changepoint vector is $\xi^+ = \xi^i \cup \{\xi^\star\}$.

The proposal ratio is given by

$$\frac{d_{k+1}}{b_k}\frac{q(\ \xi,\ \Gamma\ |\xi^+,\Gamma^+)}{q(\xi^+,\Gamma^+|\xi,\ \Gamma)}, \tag{8}$$

with

$$\frac{d_{k+1}}{b_k} = \frac{k+1}{\lambda}$$

and

$$q(\xi^+,\Gamma^+|\xi,\Gamma) = q(\xi^+|\xi)q(\Gamma^+|\Gamma,\xi,\xi^+) \tag{9}$$

where

$$q(\xi^+|\xi) = \frac{1}{((n-1)p-k)}$$

is the probability of drawing new changepoint ξ^+ when adding an extra changepoint to vector ξ, and $q(\Gamma^+)$ is the probability of drawing new topology Γ^+ when adding changepoint $\xi^\star$ to the current network with topology defined by (ξ,Γ) and

$$q(\Gamma^+|\Gamma,\xi,\xi^+) = \text{ or } \begin{array}{ll} \frac{1}{2}\ p(|\Gamma^\star| = s^\star|\Lambda)p(\Gamma^\star|s^\star) & \text{if } \Gamma^+_{h^\star_L} \neq \Gamma^+_{h^\star_R}, \\ p(|\Gamma^\star| = s^\star|\Lambda)p(\Gamma^\star|s^\star) & \text{if } \Gamma^+_{h^\star_L} = \Gamma^+_{h^\star_R}, \end{array}$$

where $p(s^\star|\Lambda) = \frac{exp(-\Lambda)}{norm_\Lambda}\frac{\Lambda^{s^\star}}{s^\star!}$ is a truncated Poisson distribution with parameter Λ (defined in Equation (???in the main text of the paper), $norm_\Lambda$ is the normalization for the truncated Poisson distribution), $p(\Gamma^\star|s^\star) = \frac{1}{\binom{s^\star}{q}} = \frac{s^\star!(q-s^\star)!}{q!}$ and $\Gamma^+_{h^\star_L}, \Gamma^+_{h^\star_R}$ are respectively the segments to the left and to the right of the new changepoint $\xi^\star$.

Furthermore $q(\xi, \Gamma|\xi^+, \Gamma^+) = q(\xi|\xi^+)q(\Gamma|\xi, \Gamma^+, \xi^+)$ is the probability of removing changepoint $\xi^\star = \xi^+ \backslash \xi$ when removing a changepoint from the changepoint vector ξ^+ where

$$q(\xi|\xi^+) = \frac{1}{(k+1)}$$

and

$$q(\Gamma|\xi, \Gamma^+, \xi^+) = \begin{array}{ll} \frac{1}{2} & \text{if } \Gamma^\star_L \neq \Gamma^\star_R, \\ \text{or} & \\ 1 & \text{if } \Gamma^\star_L = \Gamma^\star_R, \end{array}$$

as the topology of the new segment is the topology of one of the two merged segments. Finally, the proposal ratio writes,

$$\frac{d_{k+1}}{b_k} \frac{q(\ \xi,\ \Gamma\ |\xi^+, \Gamma^+)}{q(\xi^+, \Gamma^+|\xi,\ \Gamma)} = \frac{(n-1)p - k}{\lambda} \ \frac{q!}{s^\star!(q - s^\star)!} \ \frac{norm_\Lambda}{exp(-\Lambda)} \frac{s^\star!}{\Lambda^{s^\star}} \tag{10}$$

The Jacobian equals 1. Let $s^+ = s \cup \{s^\star\}$ and $\tau^+ = \tau \cup \{\tau^\star\}$ define the sets of predictors of the phases delimited by the proposed new changepoints vector ξ^+. We compute the posterior distribution ratio $\frac{\Pr(k+1, \xi^+, s^+, \tau^+|y)}{\Pr(k, \xi, s, \tau|y)}$ from equation (2). Then from proposal ratio (8), we compute,

$$r_{k,k+1}(\xi, \xi^+) = \frac{1}{(\delta^2+1)^{(s^\star+1)/2}} \frac{\left(\frac{\gamma_0}{2}\right)^{\upsilon_0/2}}{\Gamma(\frac{\upsilon_0}{2})} \frac{\Gamma_{h^\star_L}\Gamma_{h^\star_R}}{\Gamma_{h^\star}} \left(\frac{\upsilon_0 + (y^i_{h^\star})^\dagger P^i_{h^\star} y^i_{h^\star}}{2}\right)^{\frac{1}{2}(\upsilon_0 + m^i(\xi^i_{h^\star} - \xi^i_{h^\star-1}))}$$
$$\left(\frac{\upsilon_0 + (y^i_{h^\star_L})^\dagger P^i_{h^\star_L} y^i_{h^\star_L}}{2}\right)^{-\frac{1}{2}(\upsilon_0 + m^i(\xi^i_{h^\star_L} - \xi^i_{h^\star_L-1}))} \left(\frac{\upsilon_0 + (y^i_{h^\star_R})^\dagger P^i_{h^\star_R} y^i_{h^\star_R}}{2}\right)^{-\frac{1}{2}(\upsilon_0 + m^i(\xi^i_{h^\star_R} - \xi^i_{h^\star_R-1}))}. \tag{11}$$

where, for all h in $\{1, .., ki+1\}$, $\Gamma_h = \Gamma\left(\frac{\upsilon_0 + m^i(\xi^i_h - \xi^i_{h-1})}{2}\right)$. The birth of the proposed changepoint is accepted with probability,

$$\alpha_{k,k+1}(\xi, \xi^+) = \min\{1, r_{k,k+1}(\xi, \xi^+)\}. \tag{12}$$

If a birth is accepted then the sample of new parameters $(a^\star, \sigma^\star)$ from (21,22) for the new phase whose set of predictors is defined by $(s^\star, \tau^\star)$ and the state of the Markov chain becomes $(k+1, \xi^+, s^+, \tau^+, a^+, \sigma^+)$, where $a^+ = a \cup \{a^\star\}$ and $\sigma^+ = \sigma \cup \{\sigma^\star\}$. Otherwise the Markov chain remains unchanged.

3.2 Death of a changepoint

When considering the death of an existing changepoint, we first draw an existing changepoint ξ^i_h uniformly from $\{\xi^i_h\}_{1 \leq h \leq k^i}$ and collapse the neighbouring phases of changepoint ξ^i_h into a single phase. The proposed new changepoint vector is $\xi^- = \xi^i \backslash \{\xi^i_h\}$, and the set of parents for the new phase is the set Pa^i_h of parents of gene i in phase h with probability 1/2; the set Pa^i_{h+1} of parents in phase $h+1$ otherwise. The acceptance probability is then given by,

$$\alpha_{k,k-1}(\xi^i, \xi^-) = \min\{1, r^{-1}_{k^i-1,k^i}(\xi^-, \xi^i)\}. \tag{13}$$

If accepted, the neighbouring phases are collapsed into one and the state of the Markov chain becomes $(k^i - 1, \xi^-, s^-, \text{Pa}^-, \theta^-, \sigma^-)$ where the parameters with superscript "$-$" are the reduced parameter set after deletion of one phase.

3.3 Shift of a changepoint position

Moves of changepoint positions are implemented via a Metropolis update. We first choose an existing changepoint ξ_h^i uniformly from $\{\xi_h^i\}_{1\le h\le k^i}$. Then we propose to update changepoint ξ_h^i by drawing a new position $\xi_h^{i\star}$ uniformly from $[\max\{\xi_h^i - W/2, \xi_{h-1}^i+1\}, \xi_h^i - 1] \cup [\xi_h^i+1, \min\{\xi_h^i + W/2, \xi_{h+1}^i - 1\}]$, where W is a tunable window size. For short time-series, we choose $W = 2$. The new changepoint vector $\xi^\star$, obtained by replacing ξ_h^i with $\xi_h^{i\star}$, is accepted with probability $\alpha_k(\xi^i, \xi^\star) = \min\{1, r_k(\xi^i, \xi^\star)\}$ where,

$$r_k(\xi^i,\xi^\star) = \frac{\Pr(k^i,\xi^\star,s^i,\mathrm{Pa}^i|x)\ q(\xi^i|\xi^\star)}{\Pr(k^i,\xi^i,s^i,\mathrm{Pa}^i|x)\ q(\xi^\star|\xi^i)}, \tag{14}$$

and $q(\xi^\star|\xi^i)$ is the probability of drawing new changepoint $\xi^\star$ given ξ^i. The number of new changepoint vectors that can be proposed by changing the position of one element of vector ξ^i is equal to $k^iW - e$ where e is the number of impossible position changes because the gap between two successive changepoints is smaller than $W/2$.
We thus have

$$r_k(\xi^i,\xi^\star) = \left(\frac{\left(\gamma_0 + (x_h^{i\star})^\dagger P_h^{i\star} x_h^{i\star}\right)^{\upsilon_0 + m^i(\xi_h^{i\star} - \xi_{h-1}^i)} \left(\gamma_0 + (x_{h+1}^{i\star})^\dagger P_{h+1}^{i\star} x_{h+1}^{i\star}\right)^{\upsilon_0 + m^i(\xi_{h+1}^{i} - \xi_{h}^{i\star})}}{\left(\gamma_0 + (x_h^{i})^\dagger P_h^{i} x_h^{i}\right)^{\upsilon_0 + m^i(\xi_h^{i} - \xi_{h-1}^i)} \left(\gamma_0 + (x_{h+1}^{i})^\dagger P_{h+1}^{i} x_{h+1}^{i}\right)^{\upsilon_0 + m^i(\xi_{h+1}^{i} - \xi_{h}^{i})}} \right)^{1/2}$$
$$\frac{\Gamma\left(\frac{\upsilon_0 + m^i(\xi_h^{i\star} - \xi_{h-1}^i)}{2}\right)\Gamma\left(\frac{\upsilon_0 + m^i(\xi_{h+1}^{i} - \xi_{h}^{i\star})}{2}\right)}{\Gamma\left(\frac{\upsilon_0 + m^i(\xi_h^{i} - \xi_{h-1}^i)}{2}\right)\Gamma\left(\frac{\upsilon_0 + m^i(\xi_{h+1}^{i} - \xi_{h}^{i})}{2}\right)}\ \frac{kW-e}{kW-e^\star}, \tag{15}$$

where $x_h^{i\star}$ refers to the gene i expression levels observed in phase h of the changepoint vector $\xi^\star$ and $P_h^{i\star}$ is the projection matrix build from $x_h^{i\star}$ as defined in (3).

3.4 Phase Update (Updating the network topology within phases)

For regression model change moves we use a second level of RJ-MCMC computations based on the model selection procedure by Andrieu and Doucet [1,2]. When such a move is chosen, we consider regression model changes within all current phases. So for all phases h of target gene i, we successively propose three different moves: birth of a new parent, death of an existing parent or update of the regression model parameters $(\theta_{\mathrm{Pa}_h^i}, \sigma_h^i)$. The parent birth and death moves represent changes from s_h^i to s_h^i+1 or s_h^i-1 parents in the regression model. The probability for choosing theses moves are, respectively, $b_{s_h^i}$, $d_{s_h^i}$ and $v_{s_h^i}$, and satisfy $b_{s_h^i} + d_{s_h^i} + v_{s_h^i} = 1$. They are defined as follows,

$$b_{s_h^i} = c_R\ \min\left\{1, \frac{\Pr_{\overline{s}}(s_h^i+1)}{\Pr_{\overline{s}}(s_h^i)}\right\} \quad \text{and} \quad d_{s_h^i} = c_R\ \min\left\{1, \frac{\Pr_{\overline{s}}(s_h^i-1)}{\Pr_{\overline{s}}(s_h^i)}\right\}. \tag{16}$$

We take $c_R = 0.5$ so that parent birth or death moves are often proposed. This allows us to range over the set of all possible model structures. When considering a parent birth, a new parent is uniformly drawn from $\{1,...,p\}\backslash\{\mathrm{Pa}_h^i\}$ and we set the new parents subset $\mathrm{Pa}_h^{i+} = \mathrm{Pa}_h^i \cup \{j^\star\}$. A parent birth move, that is a change from Pa_h^i to Pa_h^{i+}, is accepted with acceptance probability $\alpha_{s_h^i,s_h^i+1}(\mathrm{Pa}_h^i,\mathrm{Pa}_h^{i+}) = \min\{1, r_{s_h^i,s_h^i+1}(\mathrm{Pa}_h^i,\mathrm{Pa}_h^{i+})\}$ where,

$$r_{s_h^i,s_h^i+1}(\mathrm{Pa}_h^i,\mathrm{Pa}_h^{i+}) = \frac{1}{(1+\delta^2)^{1/2}} \left(\frac{\gamma_0 + (x_h^i)^\dagger P_{\mathrm{Pa}_h^i} x_h^i}{\gamma_0 + (x_h^i)^\dagger P_{\mathrm{Pa}_h^{i+}} x_h^i}\right)^{(m^i(\xi_h^i - \xi_{h-1}^i)+\upsilon_0)/2}. \tag{17}$$

The computation of $r_{s_h^i,s_h^i+1}(\mathrm{Pa}_h^i,\mathrm{Pa}_h^{i+})$ is carried out by following Andrieu and Doucet [1]. In the same manner, a parent death move is accepted with probability $\alpha_{s_h^i,s_h^i-1}(\mathrm{Pa}_h^i,\mathrm{Pa}_h^{i-}) = \min\{1, r^{-1}_{s_{h-1}^i,s_h^i}(\mathrm{Pa}_h^{i-},\mathrm{Pa}_h^i)\}$. The update of regression model parameters is computed from equations (21) and (22) in Algorithm 1.

Algorithm 1: Phase update move

Edge birth

Choose a new parent $j^\star \sim \mathcal{U}_{\{1,\dots,p\}\setminus\{\mathrm{Pa}_h^i\}}$ and set $\mathrm{Pa}_h^{i+} = \mathrm{Pa}_h^i \cup \{j^\star\}$.
Compute $\alpha_{s_h^i, s_h^i+1}(\mathrm{Pa}_h^i, \mathrm{Pa}_h^{i+}) = min\{1, r_{s_h^i, s_h^i+1}(\mathrm{Pa}_h^i, \mathrm{Pa}_h^{i+})\}$ from (17).
Sample $u \sim \mathcal{U}_{\{0,1\}}$.
if $u \leq \alpha_{s_h^i, s_h^i+1}(Pa_h^i, Pa_h^{i+})$ **then**
 the model in phase h becomes $(s_h^i + 1, \mathrm{Pa}_h^{i+})$,
else the model remains unchanged: update parameters (a_h^i, σ_h^i) according to equations (21, 22).

Edge death

Choose one existing parent, $j^\star \sim \mathcal{U}_{\{\mathrm{Pa}_h^i\}}$, and set $\mathrm{Pa}_h^{i-} = \mathrm{Pa}_h^i \setminus \{j^\star\}$
Compute $\alpha_{s_h^i, s_h^i-1}(\mathrm{Pa}_h^i, \mathrm{Pa}_h^{i-}) = min\{1, r_{s_h^i, s_h^i-1}(\mathrm{Pa}_h^i, \mathrm{Pa}_h^{i-})\}$ from (17).
Sample $u \sim \mathcal{U}_{\{0,1\}}$.
if $u \leq \alpha_{s_h^i, s_h^i-1}(Pa_h^i, Pa_h^{i-})$ **then**
 the state i becomes $(s - 1, \mathrm{Pa}_h^{i-})$,
else the model remains unchanged: update parameters (a_h^i, σ_h^i) according to equations (21, 22).

Update regression parameters

$$(\sigma_h^i)^2 | y_h^i, \mathrm{Pa}_h^i \sim \mathcal{IG}\left(\frac{\upsilon_0 + m^i(\xi_{h+1}^i - \xi_h^i)}{2}, \frac{\gamma_0}{2} + \frac{1}{2}(x_h^i)^\dagger P_{\mathrm{Pa}_h^i} x_h^i\right) \tag{21}$$

$$a_{\mathrm{Pa}_h^i} | x_h^i, \mathrm{Pa}_h^i, \sigma_h^i \sim \mathcal{N}\left(\frac{\delta^2}{\delta^2+1}\left(D_{\mathrm{Pa}_h^i}^\dagger(x) D_{\mathrm{Pa}_h^i}(x)\right)^{-1} D_{\mathrm{Pa}_h^i}^\dagger(x)\; x_h^i,\; \frac{\delta^2(\sigma_h^i)^2}{\delta^2+1}\left(D_{\mathrm{Pa}_h^i}^\dagger(x) D_{\mathrm{Pa}_h^i}(x)\right)^{-1}\right) \tag{22}$$

3.5 Updating hyperparameters

The parameter λ is updated at each iteration of this 2-step RJ-MCMC procedure and the parameters (δ^2, Λ) are updated whenever the fourth move 'Phase update' for the network models within phases is performed. The updating of the hyperparameters is carried out as follows,

$$\lambda \mid k^i \sim \mathcal{G}a(\frac{1}{2} + k^i + \varepsilon_1, 1 + \varepsilon_2), \tag{18}$$

$$\Lambda \mid s_h^i \sim \mathcal{G}a(\frac{1}{2} + s_h^i + \varepsilon_1, 1 + \varepsilon_2), \tag{19}$$

$$\delta \mid s_h^i, \mathrm{Pa}_h^i, \theta_{\mathrm{Pa}_h^i} \sim \mathcal{IG}\left(s_h^i + \alpha_{\delta^2}, \frac{\theta_{\mathrm{Pa}_h^i} D_{\mathrm{Pa}_h^i}^\dagger(x) D_{\mathrm{Pa}_h^i}(x) \theta_{\mathrm{Pa}_h^i}}{2(\sigma_h^i)^2} + \beta_{\delta^2}\right). \tag{20}$$

4 Convergence of the algorithm

The 4th move 'Phase update' (for updating the network topology within phases) has been adapted from the RJ-MCMC procedure for model selection developed by Andrieu and Doucet [1], in which it has been established that the Markov chain generated by the iterations converges to the posterior distribution of the model parameters. This convergence occurs at a uniform geometric rate (see proof in [1]).
The three other moves for changepoint detection ('CP birth', 'CP death' and 'CP shift') are based on the same approach, performing model selection for the changepoint position vector. In practice, the number of 50,000 iterations — used in all our analyses — seems sufficient: the posterior probability $p(k|x)$ is stabilized as shown in Figure 2.

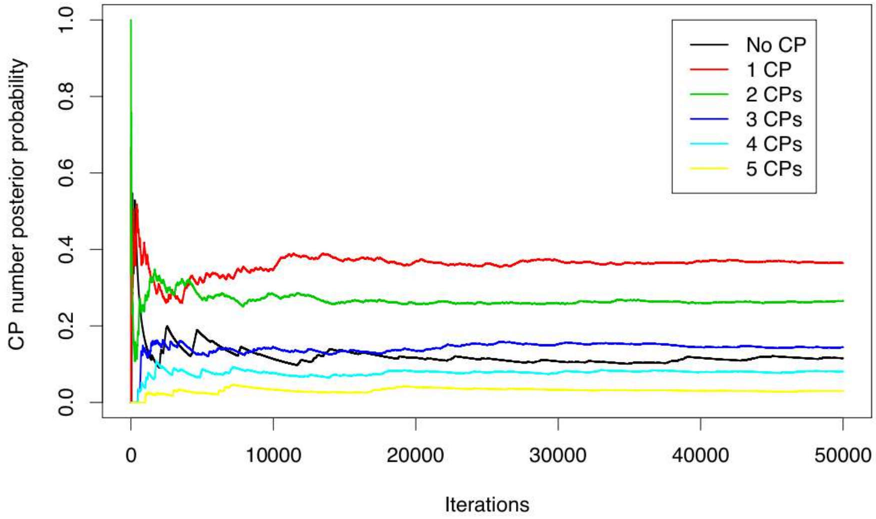

Figure 2: Instantaneous estimation of the posterior distribution of the number of changepoint k for gene *anarchist* (CG5785) in the '*Drosophila* life cycle' data by Arbeitman et al. [3].

5 Bayes factor (BF) computation

The Bayes factor [4] is a summary of the evidence provided by the data in favour of one scientific theory ($\mathcal{H}_1$), represented by a statistical model, over another $\mathcal{H}_0$. Often the null model is the complementary model to the assumption to be tested $\mathcal{H}_0 = \overline{\mathcal{H}_1}$.

The idea is to begin with data D assumed to have arisen under one of the two hypotheses $\mathcal{H}_1$ and $\mathcal{H}_0$ drawn from probability densities $P(D|\mathcal{H}_1)$ or $P(D|\mathcal{H}_0)$, respectively. Given *a priori* model probabilities $P(\mathcal{H}_1)$ and $P(\mathcal{H}_0) = 1 - P(\mathcal{H}_1)$, the data allow us to construct *a posteriori* probabilities $P(\mathcal{H}_1|D)$ and $P(\mathcal{H}_0|D) = 1 - P(\mathcal{H}_1|D)$.

These posterior probabilities are obtained through an estimation procedure. The Bayes Factor is defined as follows,

$$BF = \frac{P(D|\mathcal{H}_1)}{P(D|\mathcal{H}_0)}.$$

From Bayes's Theorem, this Bayes can be written as

$$BF = \frac{P(\mathcal{H}_1|D)}{P(\mathcal{H}_0|D)}\frac{P(\mathcal{H}_0)}{P(\mathcal{H}_1)}.$$

Following Kass and Raftery [4], a model will (i) *not* be supported with a Bayes factor below 3, (ii) be *positively* supported with a Bayes factor comprised between 3 and 20 and (iii) be *strongly* supported with a Bayes factor over 20.

We detail below the computation of the Bayes factor used in the main text. For each case, we first define the Bayes factor for the hypothesis to be tested, then we describe the computation process.

5.1 BF for the number of changepoints

Definition

Let n be the number of time points measurements, $\overline{k}$ be the maximum number of changepoints for each gene ($\overline{k} < n$) and k^i be the number of changepoints for gene i ($1 \leq i \leq p$).

For each gene i, for all $0 \leq k \leq \overline{k}$, we computed the Bayes factor related to the following assumptions:

$\mathcal{H}_0$: 'there are k changepoints for gene i in the network model' ($i.e.$ $k^i = k$)
$\mathcal{H}_1$: $\overline{\mathcal{H}_0}$

$$\begin{aligned} BF &= \frac{P(\mathcal{H}_1|D)}{P(\mathcal{H}_0|D)}\frac{P(\mathcal{H}_0)}{P(\mathcal{H}_1)} \\ &= \frac{1 - P(k^i = k|D)}{P(k^i = k|D)}\frac{P(k^i = k)}{1 - P(k^i = k)}. \end{aligned}$$

Using the marginalization over the network structures and parameters,

$$P(k^i = k|D) = \sum_{k^i,\xi^i,s^i,\mathrm{Pa}^i,\theta^i,\sigma^i} P(k^i, \xi^i, s^i, \mathrm{Pa}^i, \theta^i, \sigma^i|D)\mathbb{1}_{k^i=k},$$

the posterior probability $P(k^i = k|D)$ is given by:

$$\hat{P}(k^i = k|D) = \frac{\text{Number of iterations where the current model contains 1 changepoint exactly}}{\text{Total number of iterations}}$$

where the number of iterations refers to the number of iteration after the burn-in period.

Given that the distribution of k^i is a truncated Poisson with mean λ (truncated to the maximal number of changepoints $\overline{k}$), *i. e.*

$$\forall k \leq \overline{k}, P(k^i = k) = C(\lambda)\ \frac{\lambda^k}{k!}\ \exp(-\lambda)$$

where $C(\lambda) = \left(\exp(-\lambda)\sum_{l=0}^{\overline{k}} \frac{\lambda^l}{l!}\right)^{-1}$ is a normalization constant and $\lambda \sim \mathcal{G}a(\alpha,\beta)$, the probability of observing k changepoints for gene i writes,

$$\begin{aligned} P(k^i = k) &= \int_{\mathbb{R}_+} P(k|\lambda) f(\lambda) d\lambda \\ &= \int_{\mathbb{R}_+} C(\lambda)\ \frac{\lambda^k}{k!}\ \exp(-\lambda)\ \frac{\beta^\alpha}{\Gamma(\alpha)}\ \lambda^{\alpha-1}\ \exp(-\beta\lambda)\ d\lambda \end{aligned}$$

Computation of $P(k^i = k)$

(a) If the truncation threshold $\overline{k}$ is large (approximately starting from $\overline{k} \geq 20$), then $C(\lambda) \approx 1$ and we have,

$$\begin{aligned} P(k^i = k) &= \int_{\mathbb{R}_+} \frac{\lambda^k}{k!}\ \exp(-\lambda)\ \frac{\beta^\alpha}{\Gamma(\alpha)}\ \lambda^{\alpha-1}\ \exp(-\beta\lambda)\ d\lambda \\ &= \frac{\beta^\alpha\ \Gamma(\alpha+k)}{k!\ (\beta+1)^{\alpha+k}\ \Gamma(\alpha)} \int_{\mathbb{R}_+} \frac{(\beta+1)^{\alpha+k}}{\Gamma(\alpha+k)}\ \lambda^{(\alpha+k)-1}\ \exp\left(-(\beta+1)\lambda\right)\ d\lambda \\ &= \frac{\beta^\alpha\ \Gamma(\alpha+k)}{k!\ (\beta+1)^{\alpha+k}\ \Gamma(\alpha)} \qquad \left(\int_{\mathbb{R}_+} f_{\mathcal{G}a(\alpha+k,\beta+1)} = 1\right). \end{aligned}$$

(b) If the truncation threshold $\overline{k}$ is small (as in our case), then $C(\lambda) \neq 1$. Therefore the computation of $P(k^i = k)$ was performed numerically. Using Mathematica we computed numerically the values of the probabilities $P(k)$ for $k = 0$ to $k = \overline{k}$.

For example, for $\overline{k} = 10$, $\alpha = 1$ and $\beta = 0.5$, we obtained the distribution below.

k	0	1	2	3	4	5	...	10
$P(k^i = k)$	0.3335	0.2223	0.1484	0.0992	0.0666	0.0450	...	0.0079

5.2 BF for the changepoints position

Definition

Let $\xi^i \subseteq \{2,...,n\}$ be the changepoints position vector for gene i and $t \in \{2,...,n\}$ be a possible changepoint position, we computed the Bayes factor related to the following assumptions:

$\mathcal{H}_0$: 'There is a changepoint in position t for gene i' (*i.e.* $t \in \xi^i$).
$\mathcal{H}_1$: $\overline{\mathcal{H}_0}$

$$\begin{aligned} BF &= \frac{P(t \in \xi^i|D)}{P(t \notin \xi^i|D)}\ \frac{P(t \notin \xi^i)}{P(t \in \xi^i)} \\ &= \frac{P(t \in \xi^i|D)}{1 - P(t \in \xi^i|D)}\ \frac{1 - P(t \in \xi^i)}{P(t \in \xi^i)} \end{aligned}$$

Computation of $P(t \in \xi^i|k)$

Let $t \in \{2, ..., n\}$ be a possible changepoint position. We obtain an estimation of $P(t \in \xi^i|D)$ with the ARTIVA procedure using the marginalization over the network structures and parameters,

$$\hat{P}(t \in \xi^i|D) = \sum_{k^i, \xi^i, s^i, \mathrm{Pa}^i, \theta^i, \sigma^i} \hat{P}(k^i, \xi^i, s^i, \mathrm{Pa}^i, \theta^i, \sigma^i|D) \mathbb{1}_{t \in \xi^i},$$

and the prior probability $P(t \in \xi^i)$ is,

$$P(t \in \xi^i) = \sum_{k=0}^{\overline{k}} P(t \in \xi^i|k) P(k)$$

where $P(k)$ was computed as in Section 5.1. There are $n-1$ possible positions for a changepoint (changepoint position t and n-2 other positions) then,

$$P(t \in \xi^i|k) = \frac{\binom{1}{1}\binom{n-2}{k-1}}{\binom{n-1}{k}} = \frac{(n-2)!}{(k-1)!(n-k-1)!} \left(\frac{(n-1)!}{k!(n-k-1)!} \right)^{-1} = \frac{k}{n-1}.$$

5.3 BF for the edges (conditional on a chosen phase of the network)

Definition

Let p be the number of genes. For all gene i in $\{1, ..., p\}$, let $\xi^{i\star}_{h-1}, \xi^{i\star}_{h}$ be the changepoints delimiting a selected phase and $\mathrm{Pa}^i_{[\xi^{i\star}_{h-1}, \xi^{i\star}_{h}]}$ be the set of parent genes in the phase delimited by these changepoints. For all j in $\{1, ..., p\}$, we want to evaluate the evidence in favour of hypothesis $\mathcal{H}_0$ where,

$\mathcal{H}_0$: 'Gene j is a parent of gene i in phase delimited by $[\xi^{i\star}_{h-1}, \xi^{i\star}_{h}]$' (*i.e.* $j \in \mathrm{Pa}^i_{[\xi^{i\star}_{h-1}, \xi^{i\star}_{h}]}$).

$\mathcal{H}_1$: $\overline{\mathcal{H}_0}$

$$BF = \frac{P(j \in \mathrm{Pa}^i_{[\xi^{i\star}_{h-1}, \xi^{i\star}_{h}]}|D)}{P(j \notin \mathrm{Pa}^i_{[\xi^{i\star}_{h-1}, \xi^{i\star}_{h}]}|D)} \frac{P(j \notin \mathrm{Pa}^i_{[\xi^{i\star}_{h-1}, \xi^{i\star}_{h}]})}{P(j \in \mathrm{Pa}^i_{[\xi^{i\star}_{h-1}, \xi^{i\star}_{h}]})}$$

Computation

Let $j \in \{1, ..., p\}$ be a possible parent for gene i. We obtain an estimate of $P(j \in \mathrm{Pa}^i_{[\xi^{i\star}_{h-1}, \xi^{i\star}_{h}]}|D)$ with the ARTIVA procedure,

$$\hat{P}(j \in \mathrm{Pa}^i_{[\xi^{i\star}_{h-1}, \xi^{i\star}_{h}]}|D) = \sum_{k^i, \xi^i, \mathrm{Pa}^i, s^i, \theta^i, \sigma^i} \hat{P}(k^i, \xi^i, \mathrm{Pa}^i, s^i, \theta^i, \sigma^i|D) \mathbb{1}_{Condition1}.$$

where $Condition1$ is true when $\xi^{i\star}_{h-1}$ and $\xi^{i\star}_{h}$ are two successive changepoints in ξ^i and j is a parent gene in the phase delimited by these changepoints.

The prior probability $P(j \in \mathrm{Pa}^i_{[\xi^{i\star}_{h-1}, \xi^{i\star}_{h}]})$ is,

$$P(j \in \mathrm{Pa}^i_{[\xi^{i\star}_{h-1}, \xi^{i\star}_{h}]}) = \sum_{s=1}^{q} P(j \in \mathrm{Pa}^i_{[\xi^{i\star}_{h-1}, \xi^{i\star}_{h}]} | \; |\mathrm{Pa}^i_{[\xi^{i\star}_{h-1}, \xi^{i\star}_{h}]}| = s) P(|\mathrm{Pa}^i_{[\xi^{i\star}_{h-1}, \xi^{i\star}_{h}]}| = s)$$

where $P(|\mathrm{Pa}^i_{[\xi^{i\star}_{h-1}, \xi^{i\star}_{h}]}| = s)$ for the number of edges is computed as $P(k^i = k)$ in Section 5.1, .

References

1. Andrieu C, Doucet A: **Joint Bayesian model selection and estimation of noisy sinusoids via reversible jump MCMC**. *IEEE Trans. on Signal Processing* 1999, **47**(10):2667–2676.
2. Andrieu C, Djuri'c P, Doucet A: **Model selection by MCMC computation**. *Signal Processing* 2001, :19–37, [citeseer.ist.psu.edu/article/andrieu00model.html].
3. Arbeitman MN, Furlong F E Eand Imam, Johnson E, Null BH, Baker BS, Krasnow MA, Scott MP, Davis RW, P WK: **Gene expression during the life cycle of drosophila melanogaster.** *Science* 2002, **297**(5590):2270–2275.
4. Kass RE, Raftery AE: **Bayes Factors**. *Journal of the American Statistical Association* 1995, **90**:773–795.
