# Supplementary material for: Statistical inference of the time-varying structure of gene-regulation networks
Source: BMC Syst Biol. 2010 Sep 22;4:130. doi: 10.1186/1752-0509-4-130 (PMC2955603; doi:10.1186/1752-0509-4-130)
Supplement: Additional file 2 — Supplementary Figure S1 - Principle of the simulation study. [file 1752-0509-4-130-S2.PDF]

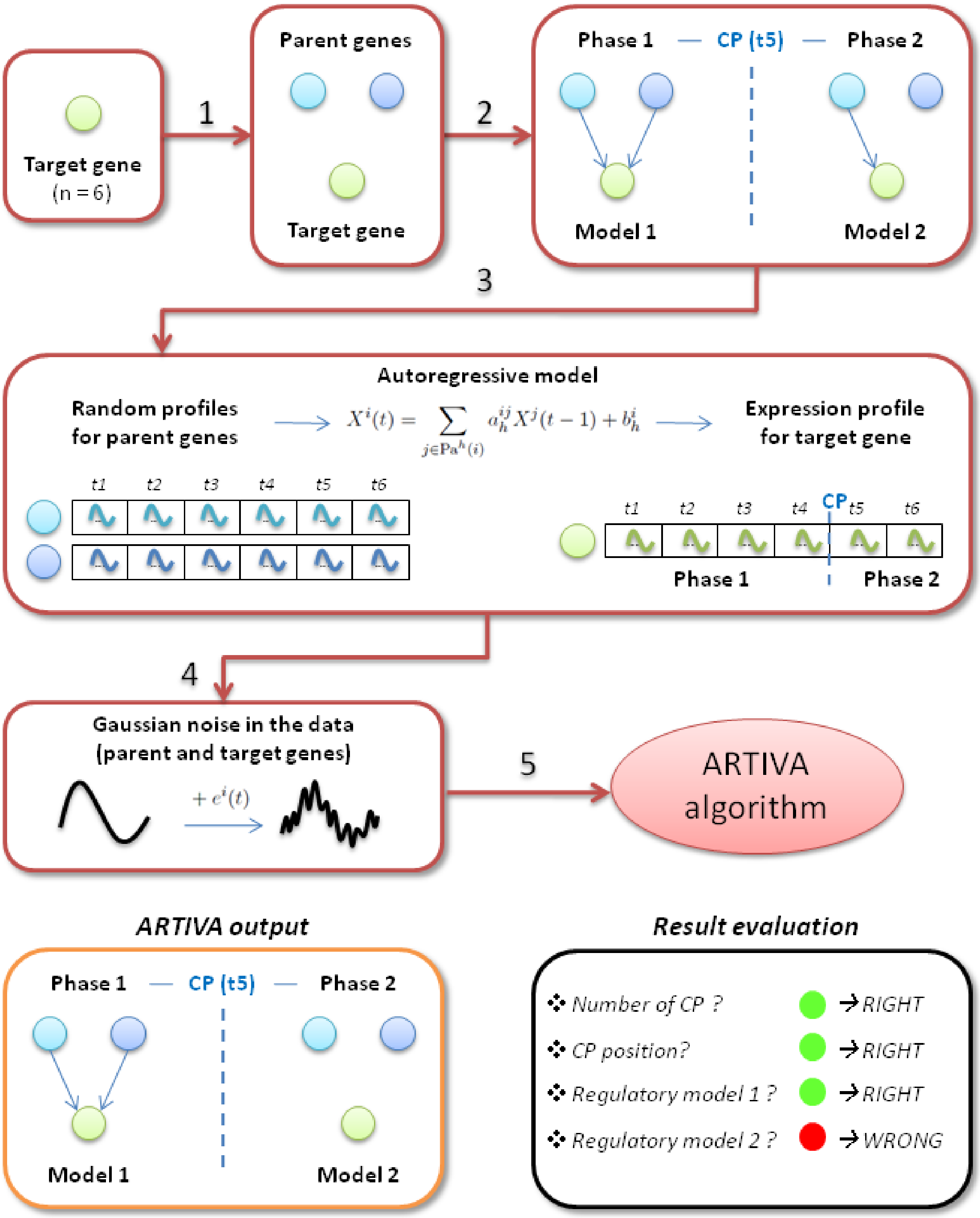

Figure S1: Principle of the simulation study. To evaluate ARTIVA performances, random networks were simulated by using the following procedure. (1) For each target gene, the number of time-points n was fixed (for instance n = 6 in this illustration) and the number of parent genes was uniformly drawn between 2 and (n - 2) (WT simulations) or n = 2 (KO simulations). The number of parent genes equals 2 in this illustration. (2) For both WT and KO simulations, the number of changepoints (CPs) was uniformly drawn between 1 and (n/4) and the CP positions were uniformly drawn between 1 and (n - 1). In the example shown here, number of CP = 1 and CP position = t5. For each phase denoted by the selected CPs, regulatory models between parent and target genes were randomly proposed. They had to be different between two successive temporal phases. (3) Expression profiles for parent genes were proposed. For that, expression values were uniformly chosen in [-2.5; 2.5] at each timepoint (WT simulations) or set to 0 or 1 over all timepoints (KO simulations). Using the regulatory models

defined in step 2 and the autoregressive model (described in Methods), expression pattern for the target gene was calculated. The interactions between parent and target genes depended, for each regulatory phase *h* and each parent gene *j*, on the values of the coefficient a^{ij}_h involved in the autoregressive model. These values were uniformly chosen in [-2; 0.1] U [0.1; 2]. (4) The data was replicated 8 (WT simulations) or 4 (KO simulations) times and Gaussian noise was finally added in the data. Several intensity of noise could be added, default values were $\mu = 0$ and $\sigma = 0.5$. (5) The entire expression dataset (parent and target gene expression profiles) obtained in step 4 was analyzed using ARTIVA algorithm and the results were compared to the initial regulatory network used in step 2. Note that to obtain the PPV and sensitivity values presented in Table 1, the entire simulation procedure (step 1 to 5 and result evaluation) was repeated 200 times. Default values for simulation parameters are summarized in Table 1 (Main Text).
