# Supplementary material for: Statistical inference of the time-varying structure of gene-regulation networks
Source: BMC Syst Biol. 2010 Sep 22;4:130. doi: 10.1186/1752-0509-4-130 (PMC2955603; doi:10.1186/1752-0509-4-130)
Supplement: Additional file 4 — Supplementary Text S2 - Supplementary results related to the 'benomyl' analyses. [file 1752-0509-4-130-S4.PDF]

**Text S2.**

**Supplementary results of hierarchical clustering and TF expression profiles of the 'benomyl' analyses**

### 1. *Choice of the number of groups for hierarchical clustering*

For a chosen number of clusters (1 to 78), the hierarchical tree was split and we calculated for each obtained cluster its intra-cluster variability (mean of expression variances at each time point). Minimal, maximal and average values are represented in Supplementary Figure S3.1 (below). In this study, 18 clusters appeared to be a relevant choice, since the maximal intra-cluster variability does not exceed 0.2, a variability value that our algorithm is able to manage (based on the simulation results).

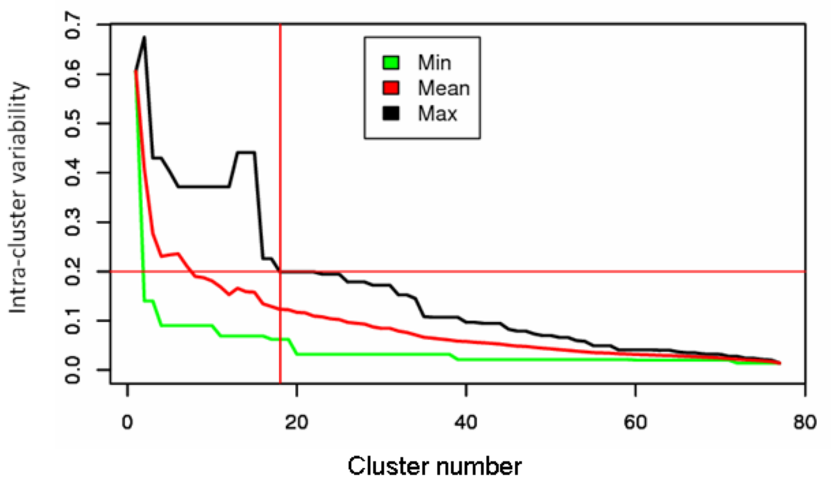


**Supplementary Figure S3.1**: Analysis of intra-cluster variability to chose the appropriate number of cluster.

### 2. *Justifications for using microarray data corresponding to diverse knockout strains*

In this study, the use of microarray data corresponding to diverse knockout strains allows us to perform a comparison of expression data between wild type and knockout strains, and hence allows a quantitative assessment of the consequence of gene deletion on global gene expression. Indeed, one must keep in mind that only the gene coding for the transcription factor YRR1 is transcriptionally

activated during the benomyl time course (see Supplementary Figure S3.2 below). The genes coding for transcription factors YAP1, PDR1 and PDR3 exhibit flat patterns that prevent the use of a classical correlation measure between expression profiles to identify parental relationships between TFs and their target genes.

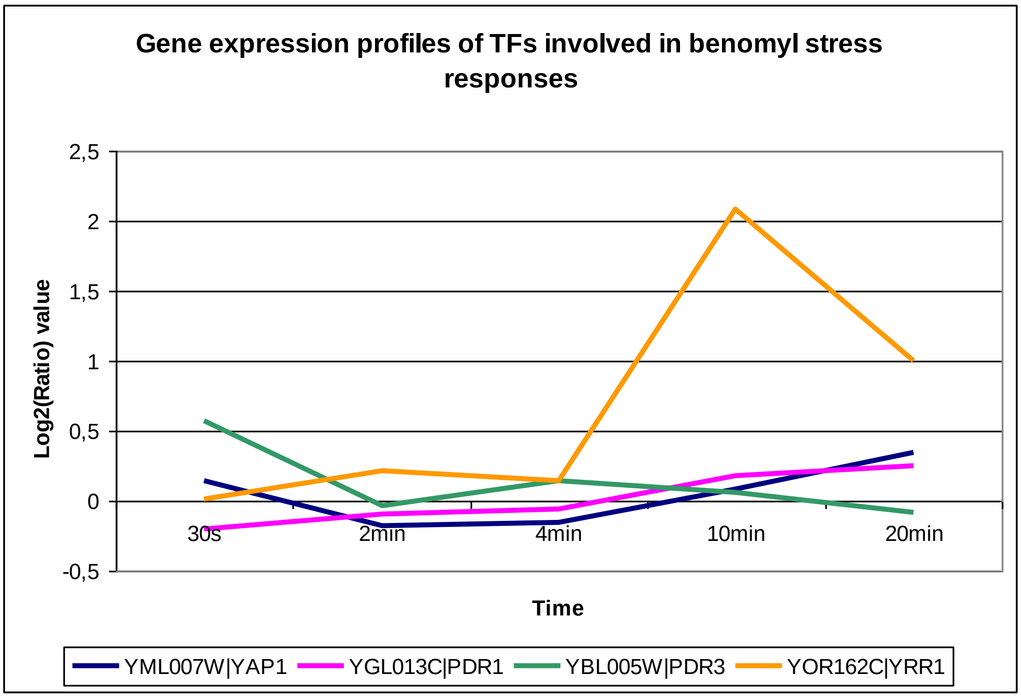


**Supplementary Figure S3.2**: Gene expression profiles of genes coding for the four transcription factors YAP1, PDR1, PDR3 and YRR1, for which the deletion effects on benomyl transcriptional response were tested.

We propose to analyze the effect of each TF knockout on the target genes expression according to the time using the multiple changepoint model described in supplementary information S1. Then for all TF $j$ in $Q = \{1, ..., 4\}$ and for all time point $t$ in {1, ..., 5}, the variable $X_t^j$ (see Eqn. 4, Main Text) is set to 0 for all $t$ in which the gene coding the TF is not deleted and set to 1 otherwise.
