# Supplementary figures and images for: Statistical inference of the time-varying structure of gene-regulation networks
Source: BMC Syst Biol. 2010 Sep 22;4:130. doi: 10.1186/1752-0509-4-130 (PMC2955603; doi:10.1186/1752-0509-4-130)

Expression  
measurements for  
the 18 clusters

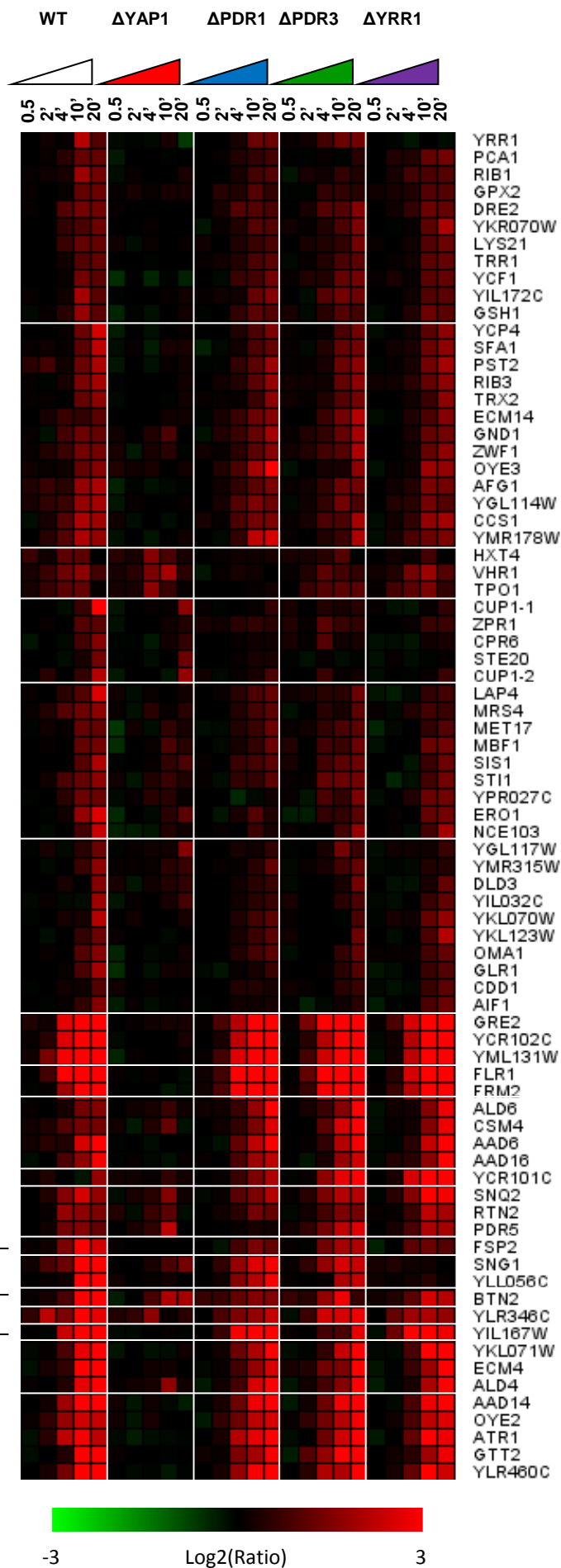

Supplement: Additional file 5 — Supplementary Figure S2 - Expression measurements for the 18 clusters used in the 'benomyl' analyses. [file 1752-0509-4-130-S5.PDF]
